# Denoise Stepwise Signals by Diffusion Model‐Based Approach

**DOI:** 10.1002/smtd.70964

**Published:** 2026-08-13

**Authors:** Xingdi Tong, Chenyu Wen

**Affiliations:** ^1^ Department of Civil and Industrial Engineering Ångströmlaboratoriet Uppsala University Uppsala Sweden; ^2^ Division of Solid‐State Electronics Department of Electrical Engineering Ångströmlaboratoriet Uppsala University Uppsala Sweden

**Keywords:** denoise, diffusion model, signal‐to‐noise ratio, single molecule detection, stepwise signal

## Abstract

Stepwise signals are ubiquitous in single‐molecule detections, where abrupt changes in signal levels typically correspond to molecular conformational changes. However, these features are inevitably obscured by noise, leading to uncertainty in estimating both signal levels and transition points. Traditional frequency‐domain filtering is ineffective for denoising stepwise signals, as edge‐related high‐frequency components strongly overlap with noise. Hidden Markov Model‐based approaches, while widely used, rely on stationarity assumptions and are not specifically designed for signal denoising. Here, we propose a diffusion model‐based algorithm for stepwise signal denoising, named the Stepwise Signal Diffusion Model (SSDM). SSDM learns the statistical structure of signals and noise. Then, it can reconstruct noise‐free signals from observations, integrating a multi‐scale convolutional network with an attention mechanism. Training data are generated by simulating stepwise signals through a Markov process with additive Gaussian noise. Across a broad range of signal‐to‐noise ratios, SSDM consistently outperforms traditional methods in both signal level reconstruction and transition point detection. Its effectiveness is further demonstrated on experimental data from single‐molecule Förster Resonance Energy Transfer and nanopore DNA translocation measurements. Overall, SSDM provides a general and robust framework for recovering stepwise signals in various single‐molecule detections and other physical systems exhibiting discrete state transitions.

## Graphical abstract

This work presents SSDM, a diffusion‐model‐based framework for denoising stepwise signals in single‐molecule measurements. By learning the statistical structure of state transitions and noise, SSDM reconstructs signal levels and identifies transition time points more accurately than conventional approaches, enabling robust analysis of signals across varied signal‐to‐noise conditions.

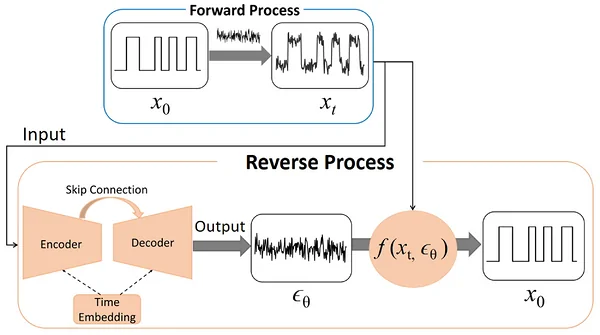

## 1 Introduction

Stepwise signals widely exist in various single‐molecule detections, in which target molecules are traced and interrogated one by one. Thus, their signals usually show stepwise shifting among different levels, instead of smoothly varying by the average effect of 10^10–^10^23^ (pM‐M) molecules in ensemble measurements. Single molecule detection technologies vary from electrical signal‐based manners, including nanopore DNA and protein sequencing [1, 2, 3, 4], translocation [5, 6], and trapping [7, 8, 9]_,_ to optical signal‐based manners, such as single‐molecule Förster Resonance Energy Transfer (sm‐FRET) [10, 11], plasmonic probes [12], and single molecule fluorescence [13, 14], and to mechanical signal‐based manners, such as atomic force microscope [15, 16] and optical/magnetic tweezers [17, 18] The stepwise shifting among different levels indicates the transition of individual molecules among several micro states [19]. Thus, the statistical features of these steps, such as the transition time point, step height, and interval between transitions, reflect molecule structures, dynamic behaviors, and interaction mechanisms [20, 21]. As can be anticipated, low signal‐to‐noise ratio (SNR) raises a major challenge in processing the stepwise signals. Severe background noise from, for example, thermal vibration [22], electromagnetic interference [23], and instrumental noise [24], leads to distortion and blurred step edges, hindering analysis accuracy [25]. Therefore, denoising is crucial for achieving accurate single‐molecule recognition, state transition detection, and kinetic parameter extraction.

Traditional frequency domain filtering methods, such as low‐pass filters, are effective only when the noise frequency components are clearly separated from those of the signal. For stepwise signals, however, transition edges contain abundant high‐frequency components that strongly overlap with the high‐frequency noise [26, 27], forcing an unavoidable trade‐off between noise suppression and transition edge preservation. Moreover, they often induce delay and attenuated amplitude, distorting the signal features. Several approaches based on statistical characteristics of signals have been proposed to address these limitations. Independent component analysis (ICA) was adopted for denoising nanopore translocation signals by exploiting correlations between current signal and the noise from the corresponding voltage stimuli [28]. However, its performance relies on strong current–voltage noise correlation, which is not always guaranteed. Cumulative sums algorithm (CUSUM) detects steps by modeling the signal as a piecewise constant function and monitoring the cumulative deviation of the signal [29].^.^However, it struggles with short events and requires user‐defined parameters.

Hidden Markov Model (HMM)‐based approaches are widely used to extract stochastic process parameters, such as state transition probabilities and dwell times [30, 31]. However, the performance is sensitive to model misspecification and to the correct selection of the number of hidden states, and they do not include an explicit signal denoising objective in the formulation [32]. Specifically, HMMs decode the most probable hidden state at each time point rather than directly reconstructing the underlying signal, and the results are sensitive to noise [33]. Practically, HMMs require a predefined number of hidden states. To guess the state number, additional trial‐and‐error‐like mechanisms are introduced [34, 35, 36], increasing computational cost [37] and reliance on manual parameter tuning. *AutoStepfinder* provides automatic step detection by iteratively minimizing the difference between the data and the fitted segments [38]. However, its performance degrades under high noise conditions and, like HMMs, it focuses on step extraction rather than denoising.

Recently, machine learning approaches have shown promise in stepwise signal processing. Nano Tree employs decision trees and the AdaBoost mechanism to fit steps in the signals [39]. Its accuracy decreases for signals with gradual transitions. Neural network‐based methods, such as convolutional autoencoder integrated in a U‐Net architecture, leverage powerful feature extraction to reconstruct noise‐free signals [40]. Nevertheless, under low SNRs, it remains challenging to achieve both signal smoothness and precise detection of transition points [41].

Given this context, to denoise stepwise signals, a natural choice is a method that does not rely on explicit state modeling and can directly learn the stochastic relationship between noise and signal from data. Denoising Diffusion Probabilistic Models (DDPM), as an emerging generative framework, is such option [42]. The model emulates thermodynamic diffusion process [43] and has achieved promising performance in denoising images, audio, and temporal signals [44, 45, 46].

Here, we propose a diffusion‐model‐based algorithm for stepwise signal denoising, named Stepwise Signal Diffusion Model, SSDM. Compared to aforementioned approaches, SSDM neither requires a clear distinction of signals and noise in frequency spectrum, nor a predefined number of states. To the best of our knowledge, this is the first time to develop a diffusion model‐based algorithm for denoising of stepwise signals. We train the SSDM on the artificially generated noisy signals by a Markov process with known additive Gaussian noise. The denoising performance is systematically evaluated under different SNR conditions. To benchmark, we quantitatively compare the denoising results with other five different methods. The comparison focuses on noise suppression capability and the accuracy of transition point recognition. Finally, to further evaluate the effectiveness and generalization capacity, we applied SSDM to experimental data from optical sm‐FRET and electrical nanopore λ‐DNA translocation measurements.

## 2 Methods

### 2.1 Stepwise Signal Diffusion Model

**FIGURE 1:**
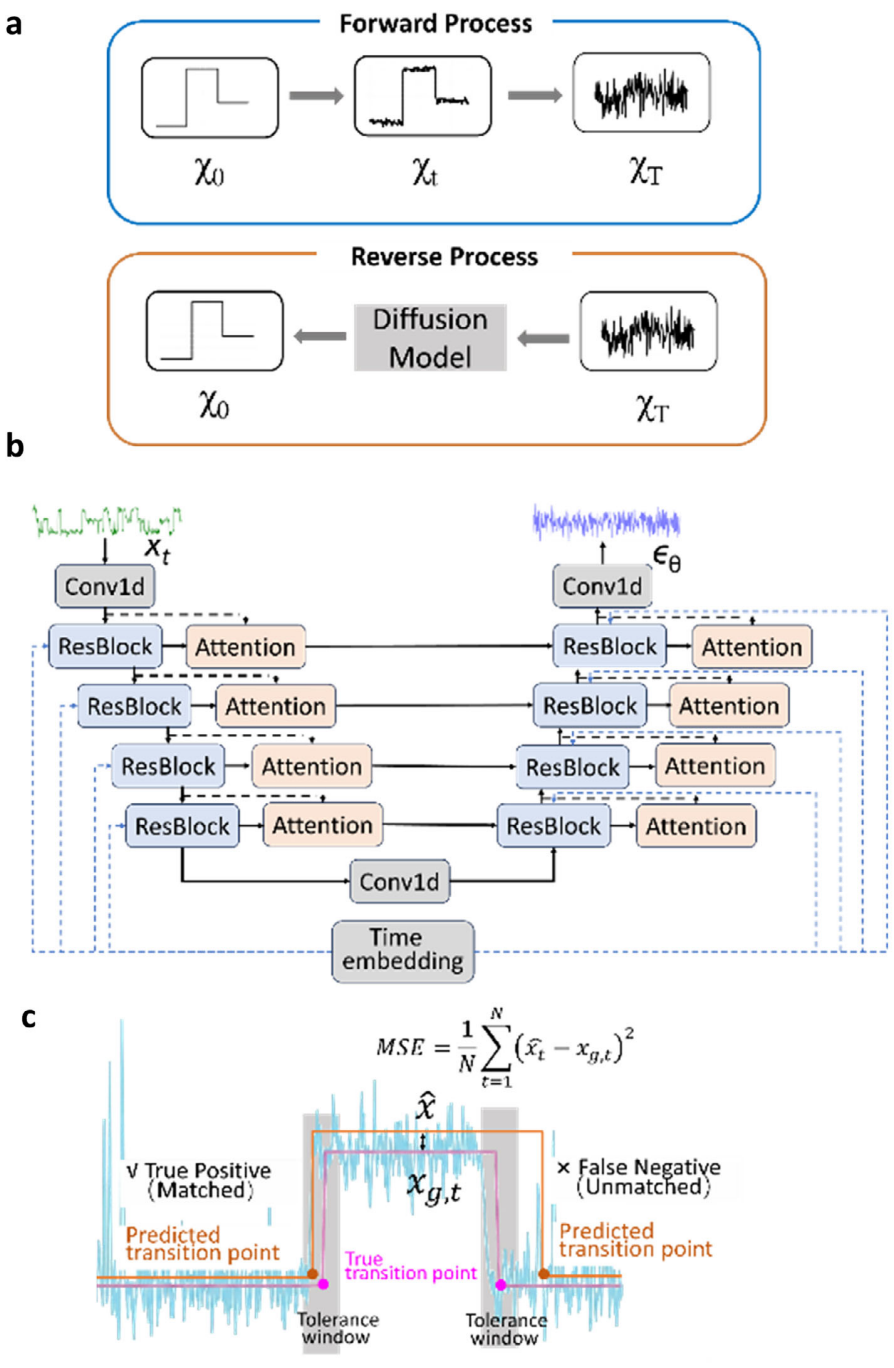
Overall structure of SSDM and the performance evaluation. (a) Schematics showing the forward and reverse diffusion processes of SSDM. (b) SSDM network architecture. (c) Schematics illustrating the performance evaluation metrics, *F*1 and *MSE*.

We adopt DDPM as the framework of SSDM, which involves forward and reverse diffusion processes, as illustrated in Figure 1a. In the forward process, a Gaussian noise is gradually injected into the original noise‐free signal *x*_0_ until the signal deforms into an extremely noisy signal *x*_T_. The forward process follows a Markov chain defined as $q\left(x_{t}|x_{t-1}\right)≔N\left(x_{t};\sqrt{1-\beta_{t}}\;x_{t-1},\beta_{t}I\right)$, which can be marginalized to a closed‐form distribution at any step *t* [42]:

(1) $$q\left(x_{t}\left|x_{0}\right.\right)=N\left(x_{t};\sqrt{{\bar{\alpha }}_{t}}x_{0},\left(1-{\bar{\alpha }}_{t}\right)I\right)$$

By reparameterization, *x*_t_ can be directly sampled as:

(2) $$x_{t}=\sqrt{{\bar{\alpha }}_{t}}x_{0}+\sqrt{1-{\bar{\alpha }}_{t}}\in \;\mathrm{with}\;\in ∼N\left(0,I\right)$$

In the reverse process, the objective of SSDM is to predict the added noise and to recover the noise‐free signal. This reverse process is defined as a Markov chain where the conditional probability from time step *t* to *t*‐1 is modeled as a Gaussian distribution parameterized by *θ*.

(3) $$p_{\theta }\left(x_{t-1}|x_{t}\right)≔N\left(x_{t-1};\mu_{\theta }\left(x_{t},t\right),\Sigma_{\theta }\left(x_{t},t\right)\right)$$

where, *x*_t_ is the noisy observation at time step *t*, *x*_t‐1_ is the predicted value at time step *t*‐1, *µ*_θ_(*x*_t_, *t*) is the mean parameter predicted by the neural network, Σ_θ_(*x*_t_, *t*) is the variance of the Gaussian distribution. *µ*_θ_ can be reparameterized as a function of the input *x_t_* and the noise *ϵ*, so we do not employ the neural network to predict *µ*_θ_ directly [42]. Our neural network is designed as a function approximator to predict the noise *ϵ*_θ_(*x*_t_, *t*) injected at each step *t*, and the mean will be derived as:

(4) $$\mu_{\theta }\left(x,t\right)=\frac{1}{\sqrt{\alpha_{t}}}\left(x_{t}-\frac{\beta_{t}}{\sqrt{\sqrt{1-{\bar{\alpha }}_{t}}}}\in_{\theta }\left(x_{t},t\right)\right)$$

Here, the noise schedule is defined by $\beta_{t}=\min\left(1-\frac{f(t)}{f(t-1)},0.999\right)\;$, $f\;\left(t\right)=\cos^{2}\left(\frac{t/T+s}{1+s}\cdot \frac{\pi }{2}\right)\;$, *s* = 0.008, and *T* = 1000. *β*_t_ is the noise schedule which employs cosine schedule introduced in the Improved DDPM framework [47], a widely adopted refinement of the linear schedule in the original DDPM. *α*_t_ = 1‐*β*_t_ and $\bar{\alpha_{t}}=\prod_{i=1}^{t}\alpha_{i}\;$ reflecting the cumulative proportion of the signal preserved during diffusion. Regarding the variance Σ_θ_(*x*_t_, *t*), we set it to a fixed schedule *β*_t_ *I*, where *I* is the identity matrix. This simplifies the sampling process by directly using the noise schedule parameters.

### 2.2 Network Architecture

To denoise stepwise signals, we need a deep learning model that can capture long‐range dependency while preserving local sudden changes. The multi‐scale architecture of U‐Net is suitable for modeling this type of data, as shown in Figure 1b. We designed a one‐dimensional (1D) convolutional neural network based on the U‐Net architecture to predict noise *ϵ*_θ_(*x*_t_, *t*). This network adopts an encoder‐decoder structure and fuses multi‐scale features through skip connections. Input is a 1D noisy stepwise signal *x_t_* with shape 1 × 1000. The embedding vector at diffusion time step *t* is also fed into the network, enabling the model to perform noise prediction at different SNR conditions. In the encoder process, the network performs a downsampling on signal at each layer, thereby increasing the temporal scope captured by subsequent convolutions. The decoder performs upsampling layer by layer with a symmetric structure as the encoder. Along the flow, it merges the corresponding encoder features into the decoding path through skip connections at each layer. This approach prevents detail loss caused by downsampling, enabling the network to simultaneously learn local fine structures and global general patterns. Each encoder and decoder layer is implemented using a residual block (Resblock) composed of convolution, normalization, activation, and skip connections. For the specific Resblock architecture, refer to Figure S1 in the Supporting Information (SI). Convolutional structures can efficiently capture local patterns but have limited capacity to model global dependencies across time steps. To address this issue, an Attention Block (refer to Figure S2) was connected after the ResBlock in each layer of both the encoder and decoder. The details of the entire network architecture can be found in Note S1.

### 2.3 Data Preparation

To train the model, we simulated stepwise signals by a 1D continuous time Markov chain. By controling the transfer rate matrix, the number of states, SNR, and the amplitude level, we generated a reproducible, scalable, and representative training dataset (see Note S2 for details). The dataset comprised balanced 2‐, 3‐, and 4‐state signals, each 1,000 time points with a normalized sampling interval. For each parameter combination, 100 independent trajectories were generated. In total, the training set contained 10,800 noise‐free signals, including 2,400 2‐state signals from 8 transition rate matrices, 3,600 3‐state signals from 12 transition rate matrices, and 4,800 4‐state signals from 16 transition rate matrices.

Noisy signals were constructed by adding Gaussian white noise to the generated signals for the train datasets, while the test datasets additionally include signals corrupted by pink (1/*f*) colored noise to evaluate robustness under different noise conditions. SNR is defined as the ratio of the minimum inter‐level difference of the signal to the peak‐to‐peak amplitude of noise (estimated as 6 times its root‐mean‐square value). Details of the noise generated can be found in Note S3. An independent test dataset was generated to evaluate generalization and robustness. It consisted of 3,600 signals with faster transitions, spanning SNRs of 0.25, 0.5, 1, 3, and 5. The test set included 40, 80, and 80 different parameter combinations for 2‐, 3‐, and 4‐state signals, respectively, with 20 trajectories per combination. A portion of the test signals was corrupted with Gaussian white noise, matching the training noise distribution, while another portion was corrupted with pink (1/*f*) noise, representing colored noise commonly encountered in experimental measurements, thereby providing a natural assessment of the robustness of the model under both matched and mismatched noise distributions. To investigate the performance of SSDM with a known number of states, we generated separate datasets containing only 2‐, 3‐, or 4‐state signals for training and test, while keeping dataset size and SNR range identical.

The experimental single‐molecule detection data are from two platforms: sm‐FRET optical signals and λ‐DNA nanopore translocation electrical signals. The sm‐FRET dataset [10] consists of 19 experimental trajectories with 100 Hz sampling rate, in total 226,100 data points. The λ‐DNA nanopore dataset [48] comprises ionic current traces recorded at different bias voltages, with 78 pM λ‐DNA dispersed in a 500 mM KCl solution, and sampled at 10 kHz. All experimental signals were normalized before denoising and rescaled back to their original amplitudes and temporal scales for analysis.

### 2.4 Model Training

The core objective of training is to enable the network to predict the noise at each step, *t*. Training follows a supervised learning procedure, with labels generated in real time during the forward diffusion process. We set the total number of diffusion steps to *T* = 1000 to ensure sufficient temporal resolution for smoothly transforming noise‐free signals into pure noise. To improve training efficiency, we employed importance sampling over diffusion steps. Specially, over time steps *t* ∈ [0, *T*] were sampled from a non‐uniform distribution *p*(*t*) ∝ exp(‐3*t*/*T*), which preferentially samples smaller *t*. Based on the Cosine Schedule in Equation (4), $\bar{\alpha_{t}}$ was calculated, and the noisy sample *x*_t_ for the current time step was generated by adding noise *ϵ* to *x*_0_ following Equation (2). It is worth noting that the noise *ϵ* serves as the training label that the network predicts. The noisy signal *x*_t_ and time embedding were fed into the model. Through feature extraction, the model ultimately predicted noise *ϵ*_θ_(*x*_t_, *t*) as its output.

### 2.5 Enhanced Loss Computation

We employed an enhanced weighted loss function to evaluate the discrepancy between the predicted noise *ϵ*_θ_(*x*_t_, *t*) and its ground‐truth *ϵ*. The Smooth L1 loss was applied to noise residual *r_i_* = *ϵ_i_*−*ϵ*_θi_ as the base loss, where 𝑖 indexes the signal sampling points:

(5) $$L_{base}\;\left(r_{i}\right)=\left\{\begin{array}{c} 0.5{r_{i}}^{2}\;,\mathrm{if}\;\left|r_{i}\right|<\;1\;\\ \;\left|r_{i}\right|-\;0.5,\mathrm{if}\;\left|r_{i}\right|\geq \;1 \end{array}\right.\;$$

This loss balances sensitivity to small errors with robustness to outliers. To jointly enhance amplitude restoration and state transition capture, we introduced a dynamic weighting mechanism, consisting of an amplitude weight and an edge weight. The amplitude weight emphasizes regions with large noise residuals, encouraging the model to suppress strong disturbances:

(6) $$W_{amp}^{\left(t\right)}=1+\lambda_{amp}\left|\in_{i}\right|$$

where, parameter *λ*_amp_ controls the sensitivity of the loss function to amplitude. The edge weight captures location and magnitude of transition edges, based on the first‐ and second‐order differences (∇*x* and ∇^2^ *x*) of the noise‐free signal *x*_0_. These differences are smoothed using a 1 × 3 convolution kernel *K* = [1/3,1/3,1/3], expanding sparse transition points into a localized penalty band:

(7) $$W_{edge}^{\left(i\right)}=1+\lambda_{edge}\left[Cpmvld\left(\nabla x+0.5\nabla^{2}x,K\right)\right]$$

where, parameter *λ*_edge_ adjusts the emphasis on the detection errors of transition points. The final training objective is defined as a weighted average over all signal trace:

(8) $$L_{total}=\frac{1}{N}\;\sum_{i=1}^{N}\left(W_{amp}^{\left(i\right)}\cdot W_{edge}^{\left(i\right)}\cdot L_{base}^{\left(i\right)}\right)$$

where, *N* is total number of signal sampling points, *W* ^(i)^_amp_ and *W* ^(i)^_edge_ are the amplitude and edge weight of the *i*‐th sampling point, and *L* ^(i)^_base_ is base loss at the *i*‐th point. Dedicated hyperparameter tuning was performed on the amplitude and edge weighting factors, *λ*_amp_ and *λ*_edge_ (see Note S4).

### 2.6 Performance Evaluation

To quantitatively evaluate the denoising performance of SSDM, we developed a comprehensive assessment framework that considers both signal amplitude recovery and accurate identification of physically meaningful state transitions, as illustrated in Figure 1c. Amplitude accuracy was measured using the mean squared error (*MSE*) between the denoised trajectory $\hat{x}$ and the signal ground‐truth *x*_gt_.

(9) $$MSE=\frac{1}{N}\;\sum_{t=1}^{N}{\left(\hat{x_{t}}-x_{gt,t}\right)}^{2}$$

Transition‐point detection accuracy was evaluated using a tolerance‐based matching criterion. A predicted transition point *t*_pred_ is considered correct if it satisfied |*t*_gt_ – *t*_pred_| ≤ *δ*, with the tolerance set to be *δ* = 2. Based on the matching results, we computed the *F1* score, which balances precision and recall:

(10) $$F1\;=2\cdot \frac{\mathrm{Precision}\cdot \mathrm{Recall}}{\mathrm{Precision}+\mathrm{Recall}}$$

with $\mathrm{Precision}\;=\frac{\mathrm{TP}}{\mathrm{TP}+\mathrm{FP}}\;$ and $\mathrm{Recall}\;=\frac{\mathrm{TP}}{\mathrm{TP}+\mathrm{FN}}\;$, where, *TP*, *FP*, and *FN* denote true positives, false positives, and false negatives, respectively. To identify the transition points, we employed amplitudes thresholds, which were predefined based on the number of states: *A*_*th*,2_ =  {0.5} for 2‐state signals, *A*_*th*,3_ =  {0.25, 0.75} for 3‐state signals, and *A*_*th*,4_ =  {0.165,  0.5,  0.83} for 4‐state signals. Each time point was assigned to a state by threshold comparison. To provide a unified performance measure, we defined a composite score combining amplitude and transition accuracy:

(11) $$\mathrm{Score}=\log\left(\frac{F1}{\mathrm{MSE}}\right)\;$$

All models were implemented using PyTorch 2.7.1 with CUDA 11.8 (cu118) and trained on a single NVIDIA L40S GPU with 48 GB memory. Training on the full dataset of 10,800 signals required approximately 23 h. The inference time of SSDM for denoising a single 1000‐point signal is approximately 5.9 s on the same GPU. Our implementation is partially modified from the open‐source PyTorch code provided in the GitHub repository “*yet‐another‐pytorch‐tutorial‐v2*” by Sungjoon Choi [49].

### 2.7 Statistical Analysis

All experimental and simulated signals were normalized to the range [0, 1] before denoising and rescaled to their original amplitude and temporal scales for subsequent analysis. No data points were excluded as outliers. Performance metrics (*MSE*, *F1*, and *Score*) are reported as descriptive statistics. In box plots, the box spans the 25th to 75th percentiles with the central line indicating the median, mean values are marked as dots, and whiskers extend from the 5th to the 95th percentiles. Data points outside the 5th–95th percentile range are not displayed as individual outliers but were retained in all statistical calculations. In bar and point plots, data are presented as mean ± standard deviation. The benchmark comparison with other methods was conducted on an independent test dataset of 3,600 signals (*n* = 3,600) across SNR levels of 0.25, 0.5, 1, 3, and 5. For the experimental validation, the sm‐FRET dataset contained 19 trajectories (*n* = 19), and the nanopore dataset comprised ionic current traces recorded at bias voltages from 300 to 800 mV, and the three‐state benchmark dataset contained 150 simulated trajectories (*n* = 150).

This study evaluates denoising performance using descriptive performance metrics (*MSE*, *F1*, and *Score*) rather than hypothesis‐testing statistics. Comparisons between methods are based on the distributions of these metrics across the test dataset, as visualized in the corresponding figures. Performance metrics (*MSE*, *F1*, and *Score*) were computed using custom Python scripts based on NumPy and pandas. Box plots and bar charts were generated using Origin2026, and other data visualizations were produced using Matplotlib.

## 3 Results and Discussion

### 3.1 Performance on the Generated Dataset

**FIGURE 2:**
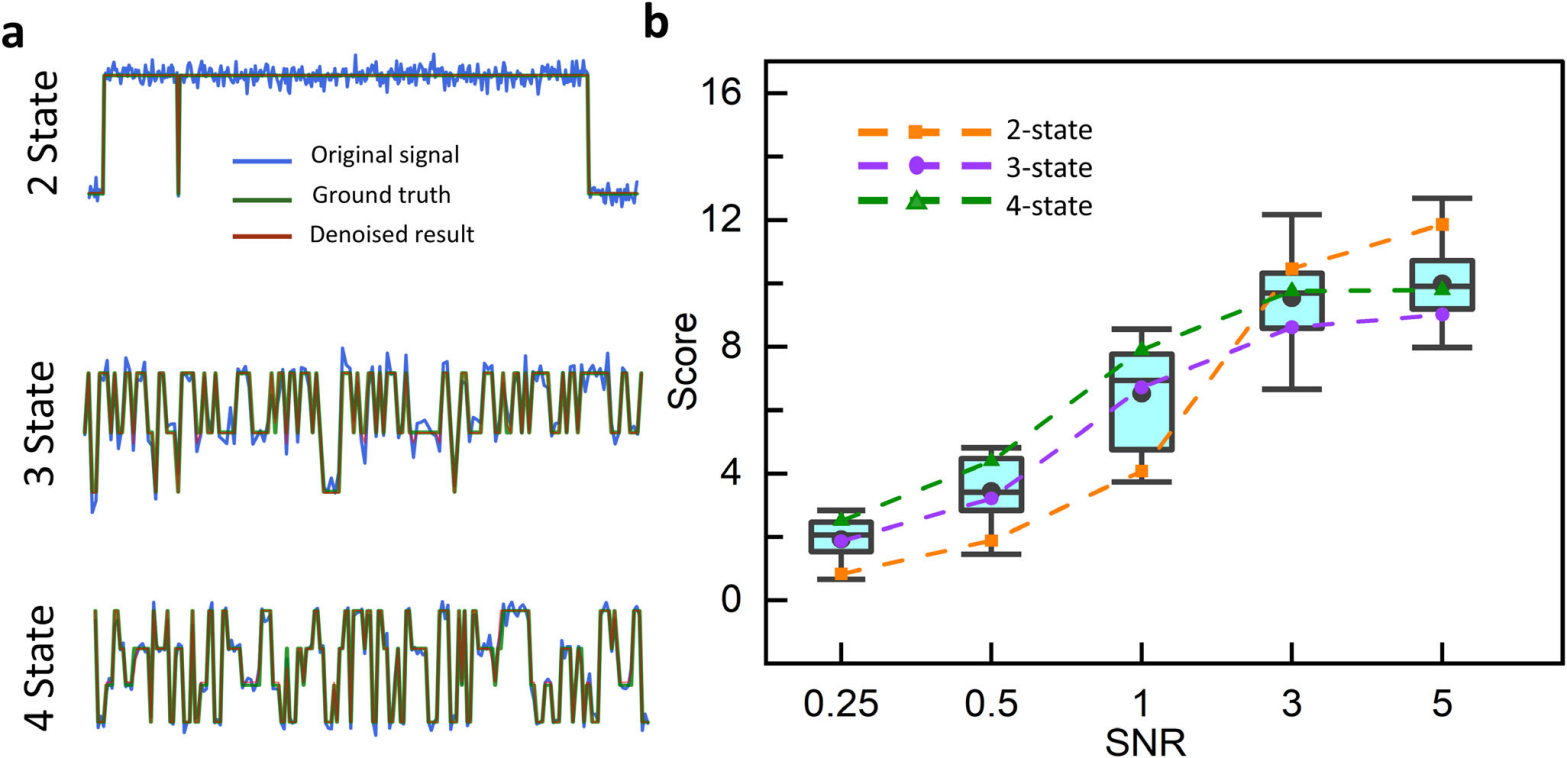
Denoising performance of SSDM for signals with different numbers of states and SNRs. (a) Typical examples of denoised signals with 2‐, 3‐, and 4‐state, respectively. The blue curves are original noisy signals with SNR = 3, green curve is the ground‐truth, and the red curves show the designed results. (b) The *Score* of the denoising performance of SSDM for signals with different SNRs. Box chart shows the results from the whole dataset. The box spans the 25th to 75th percentile, with the central line indicating the median. Mean values are marked as black dots. Whiskers extend from the 5th to the 95th percentile. The dot‐on lines represent the results of the 2‐, 3‐, and 4‐state signals from the dataset, respectively. The statistics are based on the test dataset of 3,600 signals.

SSDM was successfully trained and achieved good performance on the train dataset with *MSE* = 0.0041, *F1* = 0.96, and *Score* = 8.31. On the test dataset, the performance was *MSE* = 0.0238, *F1* = 0.81, and *Score* = 6.17. As a typical example with SNR = 3 shown in Figure 2a, the denoised signal matches the ground‐truth perfectly. It indicates the model can effectively identify the transition points and the state levels. The performance of SSDM on the test dataset depends on signal quality, that is, SNR, as shown in Figure 2b. With the increase of SNR, the impact of noise decreases, and, thus, the model gets a higher *Score* in general for all kinds of signals, that is, 2‐, 3‐, and 4‐state. The *Score* reaches a plateau at SNR = 3, indicating that further increases in the SNR have a limited contribution to improvement of denoising performance. Model performance only shows a slight variation on different kinds of signals, reflecting its adaptability to complexity of the signals. At lower SNRs (≤ 1), signals with fewer states exhibit lower *Score*, as noise fluctuations are more likely to be misidentified as state transitions.

**FIGURE 3:**
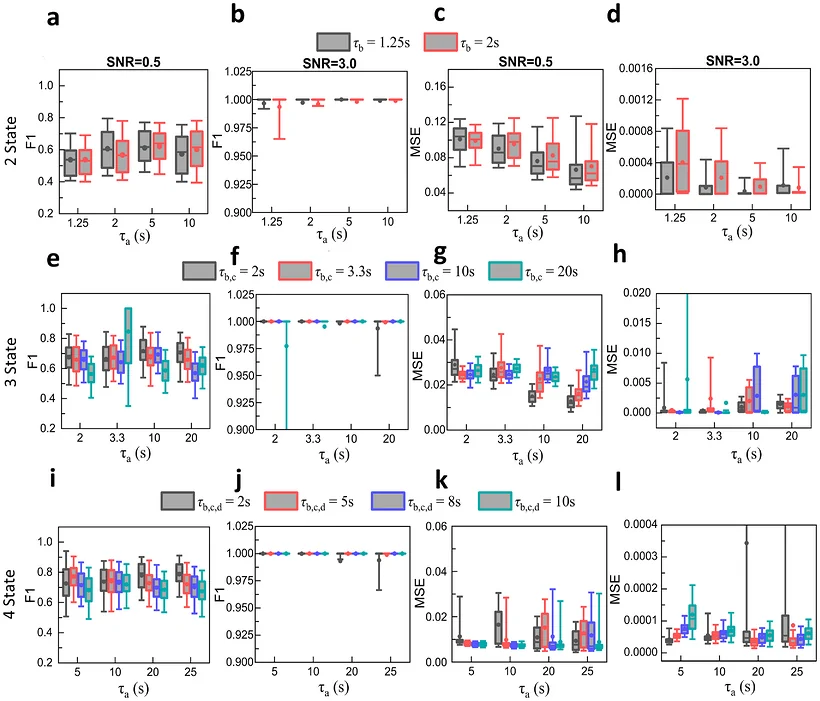
Performance of SSDM for signals with different state durations and SNRs. (a–d) *F1* and *MSE* for 2‐state signals at SNR = 0.5 and 3, respectively. (e, h) *F1* and *MSE* for 3‐state signals at SNR = 0.5 and 3, respectively. (i–l) *F1* and *MSE* for 4‐state signals at SNR = 0.5 and 3, respectively. The box spans the 25th to 75th percentiles, with the central line indicating the median. Mean values are marked as dots, and whiskers extend from the 5th to the 95th percentile.

We further analyze *F1* and *MSE* of the denoised signals with different dynamic properties, as shown in Figure 3. It was observed that when SNR > 1, *F1* approaches to one with minimal variation for all 2‐, 3‐, and 4‐state signals with different average state durations (*τ*_a,b,c,d_, the average time of the signal staying at state a, b, c, and d), as shown in Figure 3b,f,j. It indicates that SSDM performs well in both accuracy and stability for identifying transition points under higher SNR conditions. When SNR < 1, *F1* shows a general trend of a slight decline as the duration of other states increases, especially for 3‐ and 4‐state signals (see Figure 3a,e,i). It suggests that under lower SNR conditions, SSDM performs better in the identification of transition points for signals with more frequent state transitions (smaller *τ*). It can be attribute to the fact that the signals with larger *τ* have less transition events in 1000‐point trace, which offers less learning examples for the model to acquire the ability of identification of transition points.

In contrast, *MSE* demonstrates more complicated trends by varying state duration, *τ*. For 2‐state signals, *MSE* decreases in general as *τ*_a,b_ increases for all SNRs, as shown in Figure 3c,d. If a state has a long duration, the signal possesses relatively sparse transitions, which eases the state level prediction and decreases *MSE*. For 3‐ and 4‐state signals (Figure 3g,h,k,i), *MSE* is no longer determined solely by state duration, *τ*_a,b,c,d_, and more factors are involved, such as relative proportions of the durations among different states and state transition rates. *MSE* exhibits non‐monotonic trends, with a strong dependence on SNR.

### 3.2 Comparison of Generalized and Specialized Models

**FIGURE 4:**
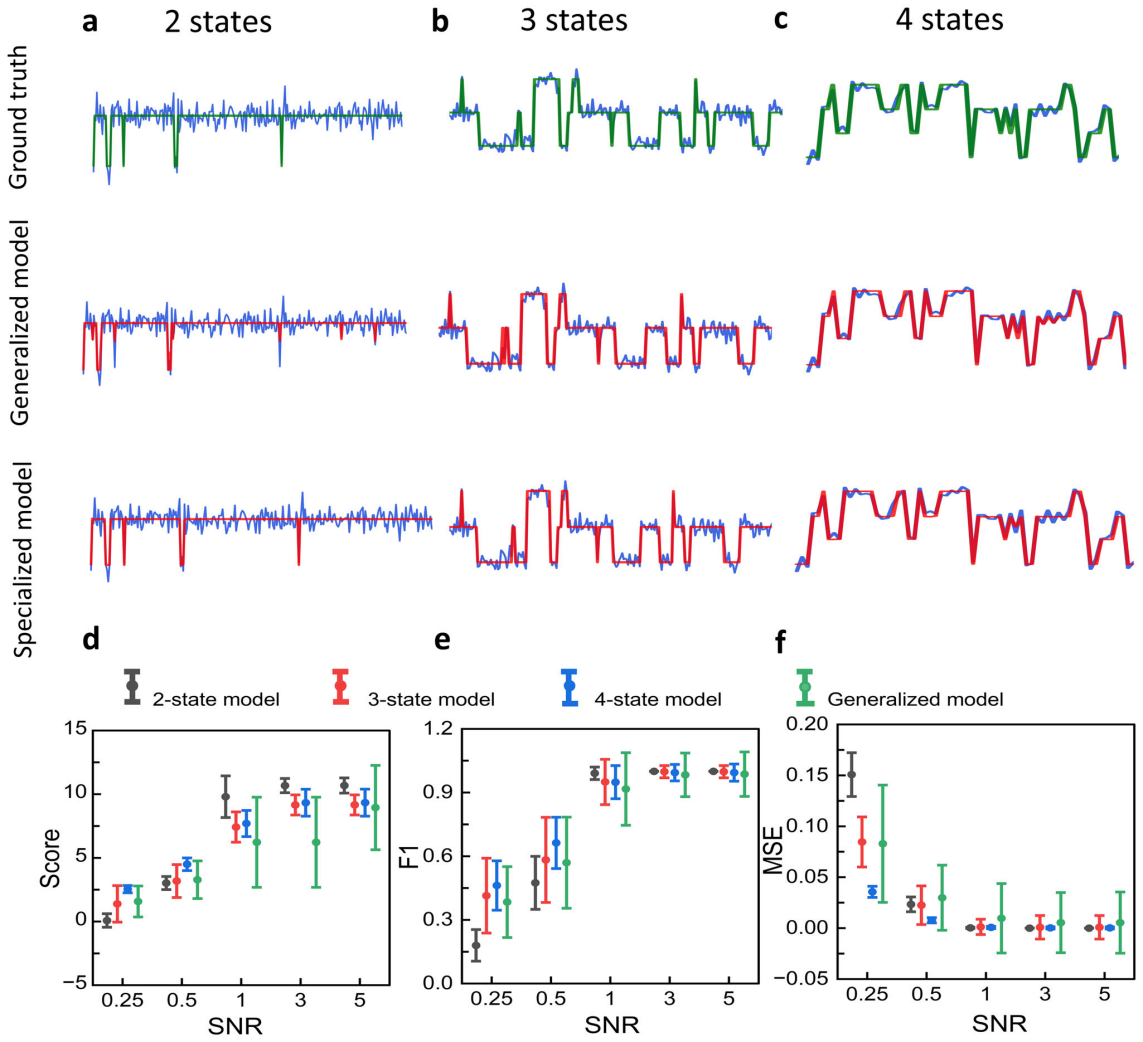
Denoising performance comparison between the specialized and generalized SSDMs. (a–c) Typical examples of ground‐truth and denoising results obtained from the specialized and generalized models for the same signal segments with 2, 3, and 4 states at SNR = 1. The blue curves are original noisy signals, green curves are the ground‐truth, and red curves show the denoised results. (d–f) The *Score*, *F1*, and *MSE* of denoising signals processed by generalized models and specialized models, respectively. The error bars denote the standard deviation, mean values are marked as dots.

To further evaluate the model performance for signals with a known number of states, we trained specialized models for 2‐, 3‐, and 4‐state, respectively, by using independently generated training and test sets for each model. The data size, ranges of corresponding parameters, and ratio among parameter combinations are the same as those used for the training and test of the generalized model in the previous section. Figure 4a–c presents representative examples comparing ground‐truth signals with denoising results obtained from the specialized and generalized models for the same signal segments with 2‐, 3‐, and 4‐state at SNR = 1, respectively. While both models effectively denoise the signals, the specialized models achieve superior performance in preserving fine details. In particular, the generalized model occasionally misrecognizes noise fluctuations as state transitions, most notably in the 2‐state case, whereas this issue is largely mitigated by the specialized models.

Overall, as the SNR increases from 0.25 to 5, the *Score* of all models show a significant increase. In general, all specialized models show a better performance than the generalized model, especially for large SNR signals, as compared in Figure 4d. In addition, the performance of specialized models has less variations indicating more stable outcomes. It suggests that if only a tiny bit more information, the number of states, is known, it can significantly help enhance the denoising performance of SSDM, since the task of processing certain kinds of signals is easier than handling all possible variations, in agreement with our intuitive understanding.

Under lower SNR conditions, particularly when SNR < 1 (Figure 4d), the 4‐state model achieves the highest *Score*, whereas the 2‐state model performs the worst. However, as the SNR increases beyond 1, the performance trend reverses. The 2‐state model exhibits a clear advantage and outperforms other models. Given that the *Score* is determined by both *F1* and *MSE*, we further examined these two metrics separately. The results indicate that when SNR ≥ 1, *F1* and *MSE* among different models are similar (Figure 4e,f), and the generalized model shows a slightly worse performance, that is, a lower *F1* and a higher *MSE*. When SNR < 1, the 2‐state model shows higher *MSE* (Figure 4f), indicating that its signal reconstruction accuracy is more heavily impacted by noise. Under low SNRs, noise causes rapid fluctuations in the signal. If a model has few states, it is difficult to distinguish these fluctuations from the real state transitions. However, models with more states can incorporate short‐duration states to describe these fluctuations, enabling more accurate signal reconstruction.

### 3.3 Comparison With Other Methods

**FIGURE 5:**
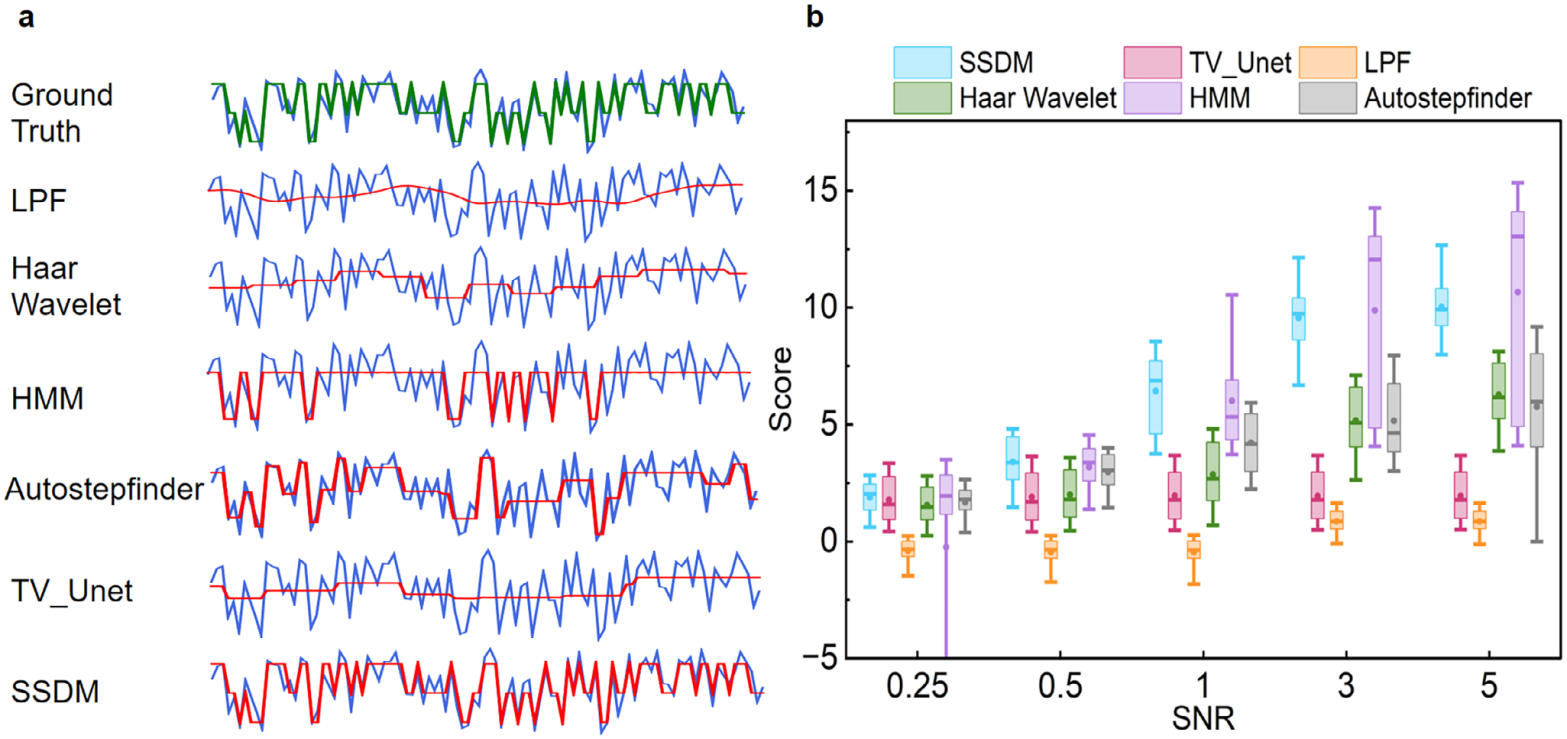
Benchmark of the denoising performance of SSDM with five comparison methods: low‐pass filter (LPF), Haar wavelet denoising, Hidden Markov Model (HMM), AutoStepfinder, and TV_Unet. (a) Typical example of a 3‐state signal segment with SNR = 0.5. Ground‐truth (green) and denoised signals (red) from each method are shown together with the original noisy signal (blue). (b) The *Score* of denoising signals with different SNRs processed by low‐pass filter, HMM, and SSDM. The box spans the 25th to 75th percentile, with the central line indicating the median. Mean values are marked as dots, and whiskers extend from the fifth to the 95th percentile. The statistics are based on the test dataset of 3,600 signals.

To better assess the performance, we benchmark the SSDM with low‐pass filters, Haar wavelet denoising, HMM, Autostepfinder, and U‐Net‐based denoising autoencoder (TV_Unet), by evaluating the results with the same metrics. We implemented a 4‐order low pass filtering to perform filtering operations on signals. The low‐pass filter is not a learning‐based method, and its only tunable parameter is the cutoff frequency. In terms of parameter selection, we performed filtering on the complete test dataset with a set of candidate cutoff frequencies, assessed *F1*, *MSE*, and the *Score* for each cutoff frequency, and selected one with the highest *Score* as the final optimized filter. For detailed implementation, refer to Note S5. A typical example of the denoised signals from the low‐pass filter with the optimized cutoff frequency is shown in Figure 5a, and the *Score* on the entire test dataset with different SNRs is shown in Figure 5b. It shows a much worse performance compared to that of the SSDM. The filtering results highly rely on the selection of the cutoff frequency, since the frequency components of the signal and noise are highly overlapped in spectrum (see Figure S2), which is an inherent physical limit of low‐pass filters and cannot be avoided.

Haar wavelet denoising was introduced as an alternative to the low‐pass filter, owing to its piecewise‐constant basis that naturally captures the structure of step‐like signals. The noisy signal is decomposed into a series of wavelet coefficients at multiple scales, in which large coefficients mostly correspond to signal transitions while small coefficients are dominated by noise. By applying a threshold to suppress the small coefficients and reconstructing the signal through inverse wavelet transform, the denoising is achieved. The threshold value is determined from the estimated noise level using the universal threshold rule. For detailed implementation, refer to Note S6. As shown in Figure 5a, the Haar wavelet denoising preserves the overall signal trend but struggles to recover sharp transitions accurately. Consequently, its Score is higher than that of the low pass filter but still substantially lower than SSDM across all SNR conditions (Figure 5b).

HMM focuses on recovering hidden state sequences, and it decodes the most probable hidden state at each time point rather than directly denoises signals. By assigning a state for each time point, HMM outputs the mean value of the corresponding state, and the output sequence looks like a denoised signal. Although it is not a model specifically designed for denoising, it can still perform the noise reduction in a certain degree. In HMMs, the number of hidden states is a critical parameter. Usually, the true number of hidden states in stepwise signals is usually unknown. To find the optimal number of states, we select the state count by iterating through possible state numbers with Bayesian Information Criterion (BIC). For detailed implementation, refer to Note S7. The same example of noisy signal treated by HMM is shown in Figure 5a. We can see that the denoised signal totally misses one state. Similarly, we employed the *Score* to evaluate the performance, as shown in Figure 5b. HMM relies on the emission probability of each state to determine which state the observation belongs to. Under conditions with lower SNR, the distribution of different states strongly overlap, which causes a significant difficulty in state recognition, and induces numerous false transitions and state missing. These result in a poor *F1* and *MSE*, so does the overall *Score*. At higher SNR, signals become clear, enabling HMM to identify state sequences more readily. However, this extreme clarity of signal introduces overfitting issues. Thus, when SNR ≥ 1, the variance in HMM *Score* increases significantly.

AutoStepfinder, a dedicated automated step detection algorithm widely used in single‐molecule analysis [38], was implemented using the official application provided by the original authors. The algorithm iteratively fits stepwise segments to the noisy signal by minimizing the residual between the data and the fitted piecewise‐constant model. A critical parameter is the acceptance ratio threshold (SMaxThreshold), which controls when the iterative fitting terminates. In our implementation, the optimal threshold was determined by visual inspection of the denoising results for each noise condition, as automated parameter selection often fails to generalize across different signal qualities. For detailed implementation, refer to Note S8. As shown in Figure 5a, AutoStepfinder captures the main transitions reasonably well at moderate SNR but tends to miss short‐duration events. Its Score is comparable to that of HMM at higher SNRs but degrades faster under low‐SNR conditions (Figure 5b). Moreover, the requirement of manual parameter tuning per signal limits its scalability when processing large datasets, where automated and parameter‐free methods become essential.

To further evaluate the contribution of the diffusion framework itself, we compared SSDM with a non‐diffusion neural network baseline, TV_Unet. It adopts the same U‐Net architecture as SSDM but without the diffusion process. It directly maps a noisy input to a noise‐free signal and is trained with Smooth L1 loss combined with a total variation regularization term. To ensure a fair architectural comparison, TV_Unet was trained on the same dataset and with the same hyperparameters as SSDM. For detailed implementation, refer to Note S9. As shown in Figure 5a, TV_Unet partially preserves the stepwise structure but exhibits residual fluctuations and inaccurate state‐level reconstruction under low‐SNR conditions. The Score is higher than traditional methods but consistently lower than SSDM (Figure 5b). Since TV_Unet and SSDM share an identical network backbone and training procedure, the performance gap can be attributed primarily to the diffusion mechanism employed in SSDM, demonstrating that the diffusion framework provides meaningful improvement beyond the network architecture itself.

In summary, SSDM achieves a higher *Score* compared to all five comparison methods. Although the *Score* of HMM is nearly as good as SSDM, it suffers much larger variations especially for SNR ≥ 1, and, thus, may deliver less stable denoising results. AutoStepfinder achieves intermediate performance but requires manual parameter tuning. TV_Unet, despite sharing the same network architecture as SSDM, falls noticeably behind, highlighting the importance of the diffusion‐based framework.

### 3.4 Validation by Experimental Data

**FIGURE 6:**
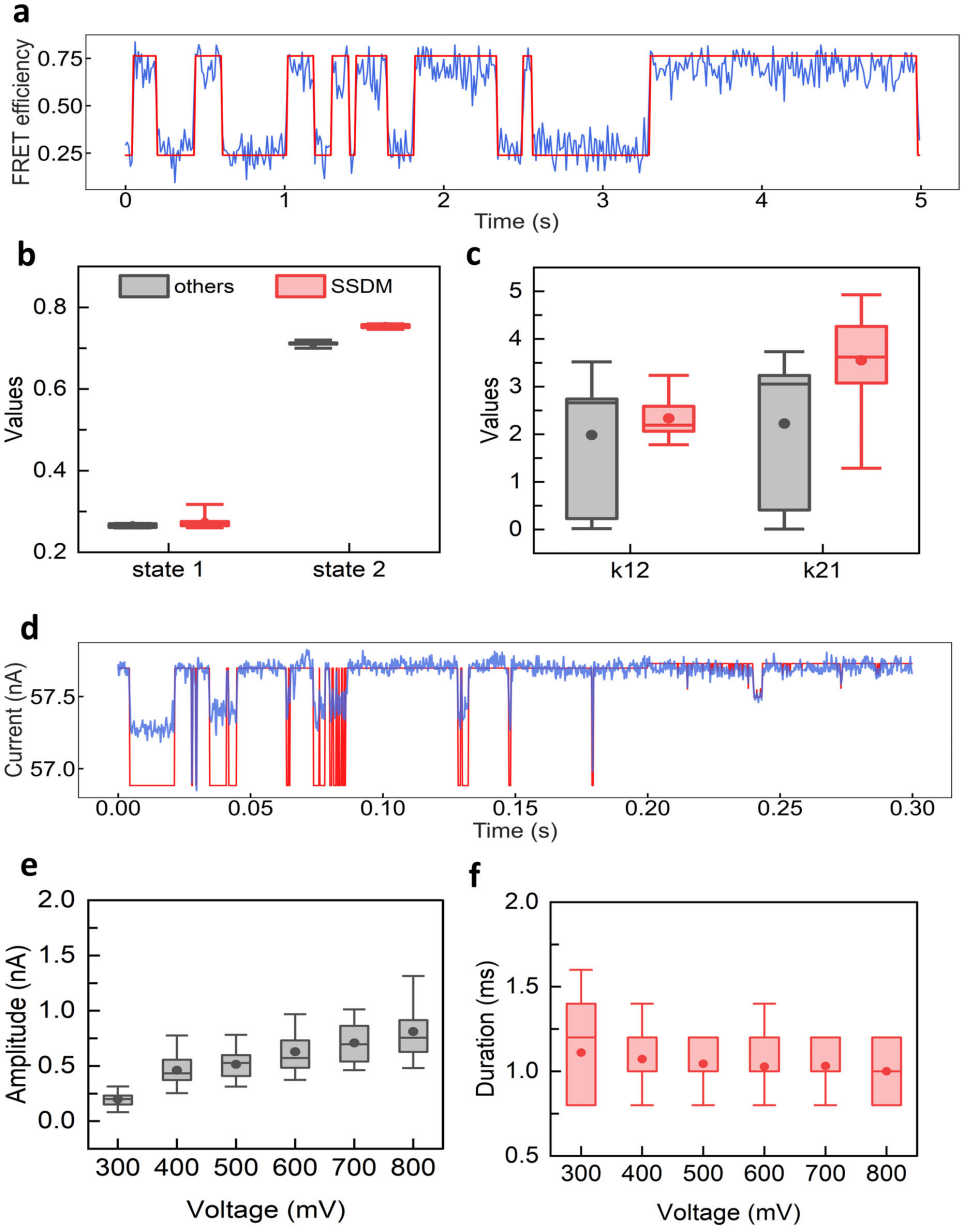
Signal processing results of the experimental data by SSDM. (a) Typical example of sm‐FRET efficiency trajectory denoised using SSDM. The blue curve is original noisy signals, the red curve shows the designed results. (b, c) Comparison of extracted state levels and kinetic parameters of smFRET state transitions obtained using SSDM and other analysis methods, respectively. (d) Typical example of nanopore λ‐DNA translocation signal processed by SSDM. The blue curve is original noisy signals, and the red curve shows the designed results. (e, f) Amplitude and duration of the λ‐DNA translocation events at different bias voltages. All box plots in the figure follow the same format: the box spans the 25th to 75th percentile, with the central line indicating the median. Mean values are marked as dots, and whiskers extend from the fifth to the 95th percentile. The sm‐FRET analysis (b,c) is based on 19 trajectories (*n* = 19). The nanopore translocation events (e, f) were extracted from ionic current traces recorded at bias voltages ranging from 300 to 800 mV.

To demonstrate the applicability of SSDM to real‐world data, we applied the trained SSDM to experimental data from sm‐FRET and nanopore λ‐DNA translocation. As a typical example shown in Figure 6a, after SSDM denoising, the FRET efficiency trace shows a clear two‐state structure. SSDM significantly reduces its background noise while honestly preserves the transition positions present in the original signal. Afterwards, each data point of the denoised signals is assigned to state 1 or 2 by referring to an amplitude threshold, which is simply selected as the midpoint value between the two state levels. Furthermore, the kinetic parameter of the two‐state transition can be extracted. The dwell times of each state coincide with exponential distributions (see Figure S3), indicating Poisson processes of the state transition in agreement with physical understanding. The corresponding kinetic rate constants *k*_12_ and *k*_21_, that is, the average transition frequency from state 1 to 2 or state 2 to 1, are extracted from the distributions.

We compared the extracted state levels and kinetic rate constants with those from other 14 different tools on the same dataset in ref [10], as shown in Figure 6b,c. It can be seen that the state levels estimated from SSDM denoised signals locate at ∼ 0.25 and ∼ 0.70, which match well with those obtained from the other tools. The kinetic rate constant *k*_12_ is close to the mean value obtained from other tools, while *k*_21_ is slightly higher than that from other tools. This deviation may come from the error of state assignment by using a simple threshold, and the results are sensitive to the threshold position. These results demonstrate the feasibility of applying SSDM to experimental sm‐FRET data, showing that the bistable structure can be recovered consistently with results from other established analysis tools.

Furthermore, we employed SSDM to denoise and analyze signals of λ‐DNA nanopore translocation at different bias voltages ranging from 300 to 800 mV. As a typical example shown in Figure 6d, after SSDM denoising, we can clearly see the translocation events without background noise fluctuations. The translocation events are extracted by applying an amplitude threshold. The amplitude and duration of each translocation event are further measured. We can clearly see from Figure 6e,f that the amplitude monotonically increases with increasing voltage, while the duration decreases as voltage increases. This trend complies with the physical understanding: a higher voltage generates a larger ionic current so does the translocation spikes; a higher voltage drives the translocation of DNA faster through the nanopore, indicating a short duration. In addition, the results are consistent with those from other algorithms [48, 50]. These results illustrate the applicability of SSDM to nanopore translocation data, where the denoised signals exhibit physically reasonable amplitude and duration trends with respect to bias voltage.

To further assess the applicability of SSDM to more complex multi‐state dynamics, we additionally evaluated it on a three‐state simulated sm‐FRET dataset from the same benchmark study [10]. Although experimental three‐state sm‐FRET datasets with known ground‐truth kinetic parameters are difficult to obtain, the simulated dataset provides well‐defined state levels and transition rates, enabling quantitative validation. SSDM successfully resolved the three FRET states and recovered all six inter‐state kinetic rate constants in good agreement with the ground truth, demonstrating its capability to preserve both state and kinetic information in more complex systems. Furthermore, the three‐state benchmark contains non‐uniformly distributed state levels, indicating that SSDM can generalize beyond the uniformly spaced state configurations used in the quantitative benchmarks of Sections 3.1–3.3. Detailed results are presented in Figure S4.

## 4 Discussion

SSDM demonstrates superior denoising performance on stepwise signals, particularly in its ability to preserve sharp state transitions while effectively suppressing noise. To further adapt and optimize the model for diverse real‐world applications, several practical considerations should be taken into account.

The length of input signal segment directly affects the inference time in the reverse diffusion process. In current implementation, SSDM operates on fixed 1000‐point signal segments. However, this length can be adjusted according to signal properties in different applications, allowing a balance among segment length, denoising performance, and computation efficiency.

Furthermore, the standard DDPM framework adopts Gaussian white noise as the noise model during training. As a result, although SSDM remains robust on test datasets that also contain pink noise, its denoising is inherently optimized for white‐noise statistics. For signals with noise characteristics that deviate substantially from this assumption, model performance can be further improved by generating training datasets that include corresponding noise.

For more challenging scenarios, such as signals with extremely low SNR, a large number of states, and various state levels, additional optimization of SSDM hyperparameter is necessary. The trade‐off between accurate detection of transition points and fitting state levels can be controlled by tuning the loss function. Training efficiency may also be improved by optimizing the evaluation metrics.

For applications demand Real‐time data processing, inference latency becomes a critical factor. In such cases, advanced versions of diffusion‐based models may be adopted, such as Denoising Diffusion Implicit Models and other accelerated variants [51]. Moreover, when deployment on embedded low‐power hardware platforms is required, a careful balance between model complexity and lightweight design should be considered.

It is worth noting that the term “denoising” in this work refers to the removal of all noise components from the observed signal, including both extrinsic instrumental noise and intrinsic molecular fluctuations. Extending SSDM to selectively remove only extrinsic noise sources, while preserving intrinsic dynamics relevant for free‐energy‐landscape reconstruction [52], represents a promising direction for future work.

## 5 Conclusion

In this work, we propose SSDM, a diffusion‐based framework for denoising stepwise signals. The model adopts an iterative denoising strategy that combines a U‐Net architecture with attention mechanisms to effectively capture both local features and long‐range dependencies. To quantitatively assess denoising performance for stepwise signals, we introduce a set of task‐specific metrics that jointly evaluate state‐level reconstruction accuracy and state‐transition identification. Through tailored loss functions and evaluation strategies, SSDM achieves improved denoising accuracy while enhancing the reliability of state and transition detection. Benchmark results demonstrate that SSDM substantially reduces noise from stepwise signals even under severe noise conditions with SNR < 1. Furthermore, specialized models trained for 2‐, 3‐, and 4‐state signals outperform a generalized model, indicating that even limited prior knowledge of the number of states can significantly enhance performance. Compared to a range of traditional and learning‐based methods including low‐pass filtering, Haar wavelet denoising, HMM, AutoStepfinder, and a non‐diffusion U‐Net baseline, SSDM achieves superior performance in terms of the overall denoising *Score*. The effectiveness of SSDM is further validated using real experimental data from sm‐FRET and nanopore DNA translocation measurements. The denoised results are consistent with those obtained using other established methods and align with physical expectations, demonstrating the strong generalization capability of SSDM. Overall, SSDM provides a robust and flexible framework for stepwise signal denoising and shows significant promise for addressing complex signal processing challenges across a wide range of applications.

## Conflicts of Interest

The authors declare no conflicts of interest.

## Supporting information




**Supporting File**: smtd70964‐sup‐0001‐SuppMat.pdf.

## Data Availability

The generated data sets for all the combinations of the parameters are available at https://doi.org/10.5281/zenodo.18432190. The code for all SSDMs used in this work is available at https://doi.org/10.5281/zenodo.20397276.

## References

[1] A. Dorey and S. Howorka , “Nanopore DNA Sequencing Technologies and Their Applications Towards Single‐Molecule Proteomics,” Nature Chemistry 16, no. 3 (2024): 314–334, 10.1038/s41557-023-01322-x. 38448507

[2] J. Ritmejeris , X. Chen , and C. Dekker , “Single‐Molecule Protein Sequencing With Nanopores,” Nature Reviews Bioengineering 3, no. 4 (2025): 303–316, 10.1038/s44222-024-00260-8.

[3] Y. Wang , Y. Zhao , A. Bollas , Y. Wang , and K. F. Au , “Nanopore Sequencing Technology, Bioinformatics and Applications,” Nature Biotechnology 39, no. 11 (2021): 1348–1365, 10.1038/s41587-021-01108-x. PMC8988251 34750572

[4] Y.‐L. Ying , Z.‐L. Hu , S. Zhang , et al., “Nanopore‐Based Technologies Beyond DNA Sequencing,” Nature Nanotechnology 17, no. 11 (2022): 1136–1146, 10.1038/s41565-022-01193-2. 36163504

[5] F. Meng , X. Li , N. Zou , and X. Wang , “Protein Profiling by Nanopore‐Based Technology,” Analytical Chemistry 97, no. 19 (2025): 10110–10125, 10.1021/acs.analchem.5c00992. 40326163

[6] J. Houghtaling , C. Ying , O. M. Eggenberger , et al., “Estimation of Shape, Volume, and Dipole Moment of Individual Proteins Freely Transiting a Synthetic Nanopore,” ACS Nano 13, no. 5 (2019): 5231–5242, 10.1021/acsnano.8b09555. 30995394

[7] P. Tripathi , A. Firouzbakht , M. Gruebele , and M. Wanunu , “Threading Single Proteins Through Pores to Compare Their Energy Landscapes,” Proceedings of the National Academy of Sciences 119, no. 39 (2022): 2202779119, 10.1073/pnas.2202779119. PMC9522335 36122213

[8] C. Wen , E. Bertosin , X. Shi , C. Dekker , and S. Schmid , “Orientation‐Locked DNA Origami for Stable Trapping of Small Proteins in the Nanopore Electro‐Osmotic Trap,” Nano Letters 23, no. 3 (2023): 788–794, 10.1021/acs.nanolett.2c03569. PMC9912335 36507712

[9] S. Schmid , P. Stömmer , H. Dietz , and C. Dekker , “Nanopore Electro‐Osmotic Trap for the Label‐Free Study of Single Proteins and Their Conformations,” Nature Nanotechnology 16, no. 11 (2021): 1244–1250, 10.1038/s41565-021-00958-5. 34462599

[10] M. Götz , A. Barth , S. S.‐R. Bohr , et al., “A Blind Benchmark of Analysis Tools to Infer Kinetic Rate Constants From Single‐Molecule FRET Trajectories,” Nature Communications 13 (2022): 5402, 10.1038/s41467-022-33023-3. PMC9474500 36104339

[11] B. Vermeer and S. Schmid , “Can DyeCycling Break the Photobleaching Limit in Single‐Molecule FRET?,” Nano Research 15, no. 11 (2022): 9818–9830, 10.1007/s12274-022-4420-5. PMC9101981 35582137

[12] A. B. Taylor and P. Zijlstra , “Single‐Molecule Plasmon Sensing: Current Status and Future Prospects,” ACS Sensors 2, no. 8 (2017): 1103–1122, 10.1021/acssensors.7b00382. PMC5573902 28762723

[13] S. Ray , J. R. Widom , and N. G. Walter , “Life Under the Microscope: Single‐Molecule Fluorescence Highlights the RNA World,” Chemical Reviews 118, no. 8 (2018): 4120–4155, 10.1021/acs.chemrev.7b00519. PMC5918467 29363314

[14] J. Chu , A. Ejaz , K. M. Lin , et al., “Single‐Molecule Fluorescence Multiplexing by Multi‐Parameter Spectroscopic Detection of Nanostructured FRET Labels,” Nature Nanotechnology 19, no. 8 (2024): 1150–1157, 10.1038/s41565-024-01672-8. PMC11329371 38750166

[15] M. Carrion‐Vazquez , A. F. Oberhauser , S. B. Fowler , et al., “Mechanical and Chemical Unfolding of a Single Protein: A Comparison,” Proceedings of the National Academy of Sciences 96, no. 7 (1999): 3694–3699, 10.1073/pnas.96.7.3694. PMC22356 10097099

[16] M. Mora , R. Tapia‐Rojo , and S. Garcia‐Manyes , “Unfolding and Refolding Proteins Using Single‐Molecule AFM,” in Single Molecule Analysis, ed. I. Heller , D. Dulin , and E. J. G. Peterman , (Springer US, 2024), 339–354. Methods in Molecular Biology, 10.1007/978-1-0716-3377-9_16. 37824012

[17] C. J. Bustamante , Y. R. Chemla , S. Liu , and M. D. Wang , “Optical Tweezers in Single‐Molecule Biophysics,” Nature Reviews Methods Primers 1, no. 1 (2021): 25, 10.1038/s43586-021-00021-6. PMC8629167 34849486

[18] R. Tapia‐Rojo , M. Mora , and S. Garcia‐Manyes , “Single‐Molecule Magnetic Tweezers to Probe the Equilibrium Dynamics of Individual Proteins at Physiologically Relevant Forces and Timescales,” Nature Protocols 19, no. 6 (2024): 1779–1806, 10.1038/s41596-024-00965-5. PMC7616092 38467905

[19] A. Janshoff , M. Neitzert , Y. Oberdörfer , and H. Fuchs , “Force Spectroscopy of Molecular Systems—Single Molecule Spectroscopy of Polymers and Biomolecules,” Angewandte Chemie International Edition 39, no. 18 (2000): 3212–3237, 10.1002/1521-3773(20000915)39:18<3212::AID-ANIE3212>3.0.CO;2-X. 11028062

[20] Y. Ishii and T. Yanagida , “How Single Molecule Detection Measures the Dynamic Actions of Life,” HFSP Journal 1, no. 1 (2007): 15–29, 10.2976/1.2723643/10.2976/1. PMC2645551 19404457

[21] Y. Li , C. Yang , and X. Guo , “Single‐Molecule Electrical Detection: A Promising Route Toward the Fundamental Limits of Chemistry and Life Science,” Accounts of Chemical Research 53, no. 1 (2020): 159–169, 10.1021/acs.accounts.9b00347. 31545589

[22] M. Wang , D. J. Perez‐Morelo , G. Ramer , et al., “Beating Thermal Noise in a Dynamic Signal Measurement by a Nanofabricated Cavity Optomechanical Sensor,” Science Advances 9, no. 11 (2023): adf7595, 10.1126/sciadv.adf7595. PMC10017032 36921059

[23] M. Hardage and P. D. Henry , "Electromagnetic Interference (EMI)," in Implantable Defibrillator Therapy: A Clinical Guide, eds. A. Pacifico , G. H. Bardy , M. Borggrefe , P. D. Henry , F. E. Marchlinski , A. Natale , and B. I. Wilkoff , (Springer US, 2002): 325–350, 10.1007/978-1-4615-1055-0_13.

[24] J. C. Driggers , J. Harms , and R. X. Adhikari , “Subtraction of Newtonian Noise Using Optimized Sensor Arrays,” Physical Review D (2012): 102001, 10.1103/PhysRevD.86.102001.

[25] B. C. Carter , M. Vershinin , and S. P. Gross , “A Comparison of Step‐Detection Methods: How Well Can You Do?,” Biophysical Journal 94, no. 1 (2008): 306–319, 10.1529/biophysj.107.110601. PMC2134886 17827239

[26] D. Strong and T. Chan , “Edge‐Preserving and Scale‐Dependent Properties of Total Variation Regularization,” Inverse Problems 19, no. 6 (2003): S165–S187, 10.1088/0266-5611/19/6/059.

[27] L. I. Rudin , S. Osher , and E. Fatemi , “Nonlinear Total Variation Based Noise Removal Algorithms,” Physica D: Nonlinear Phenomena 60, no. 1 (1992): 259–268, 10.1016/0167-2789(92)90242-F.

[28] Q. D. Y. Ma , S. Nie , R. Jin , J. Yuan , L. Tang , and L. Wu , “Robust Noise Separation and Denoising of Ionic Current Signals for Nanopore Sensors Based on Independent Component Analysis,” Journal of Physical Chemistry Letters 16, no. 41 (2025): 10612–10620, 10.1021/acs.jpclett.5c02534. 41047590

[29] C. Raillon , P. Granjon , M. Graf , L. J. Steinbock , and A. Radenovic , “Fast and Automatic Processing of Multi‐Level Events in Nanopore Translocation Experiments,” Nanoscale 4, no. 16 (2012): 4916, 10.1039/c2nr30951c. 22786690

[30] L. S. Milescu , A. Yildiz , P. R. Selvin , and F. Sachs , “Extracting Dwell Time Sequences From Processive Molecular Motor Data,” Biophysical Journal 91, no. 9 (2006): 3135–3150, 10.1529/biophysj.105.079517. PMC1614497 16905607

[31] J. Bechhoefer , “Hidden Markov Models for Stochastic Thermodynamics,” New Journal of Physics 17, no. 7 (2015): 075003, 10.1088/1367-2630/17/7/075003.

[32] L. R. Rabiner , “A Tutorial on Hidden Markov Models and Selected Applications in Speech Recognition,” Proceedings of the IEEE 77, no. 2 (1989): 257–286, 10.1109/5.18626.

[33] D. Esguerra and S. B. Munch , “Accounting for Observation Noise in Equation‐Free Forecasting: The Hidden‐Markov S‐Map,” Methods in Ecology and Evolution 15, no. 8 (2024): 1347–1359, 10.1111/2041-210X.14337.

[34] M. Greenfeld , D. S. Pavlichin , H. Mabuchi , and D. Herschlag , “Single Molecule Analysis Research Tool (SMART): An Integrated Approach for Analyzing Single Molecule Data,” PLoS ONE 7, no. 2 (2012): 30024, 10.1371/journal.pone.0030024. PMC3282690 22363412

[35] S. A. McKinney , C. Joo , and T. Ha , “Analysis of Single‐Molecule FRET Trajectories Using Hidden Markov Modeling,” Biophysical Journal 91, no. 5 (2006): 1941–1951, 10.1529/biophysj.106.082487. PMC1544307 16766620

[36] S. Schmid , M. Götz , and T. Hugel , “Single‐Molecule Analysis Beyond Dwell Times: Demonstration and Assessment in and out of Equilibrium,” Biophysical Journal 111, no. 7 (2016): 1375–1384, 10.1016/j.bpj.2016.08.023. PMC5052450 27705761

[37] J. Pohle , R. Langrock , F. M. van Beest , and N. M. Schmidt , “Selecting the Number of States in Hidden Markov Models: Pragmatic Solutions Illustrated Using Animal Movement,” Journal of Agricultural, Biological and Environmental Statistics 22, no. 3 (2017): 270–293, 10.1007/s13253-017-0283-8.

[38] L. Loeff , J. W. J. Kerssemakers , C. Joo , and C. Dekker , “AutoStepfinder: A Fast and Automated Step Detection Method for Single‐Molecule Analysis,” Patterns 2, no. 5 (2021): 100256, 10.1016/j.patter.2021.100256. PMC8134948 34036291

[39] D. Wadhwa , P. Mensing , J. Harden , P. Branco , V. Tabard‐Cossa , and K. Briggs , “Nano Trees: Nanopore Signal Processing and Sublevel Fitting Using Decision Trees,” Digital Discovery 4, no. 7 (2025): 1743–1750, 10.1039/d5dd00060b.

[40] M. Tsutsui , T. Takaai , K. Yokota , T. Kawai , and T. Washio , “Deep Learning‐Enhanced Nanopore Sensing of Single‐Nanoparticle Translocation Dynamics,” Small Methods 5, no. 7 (2021): 2100191, 10.1002/smtd.202100191. 34928002

[41] V. A. Raj , T. Parupudi , A. Thalengala , and S. G. Nayak , “A Comprehensive Review of Deep Learning Models for Denoising EEG Signals: Challenges, Advances, and Future Directions,” Discover Applied Sciences 7, no. 11 (2025): 1268, 10.1007/s42452-025-07808-2.

[42] J. Ho , A. Jain , and P. Abbeel , “Denoising Diffusion Probabilistic Models,” in NIPS″20: Proceedings of the 34th International Conference on Neural Information Processing System (2020).

[43] M. N. Yeğin and M. F. Amasyalı , “Generative Diffusion Models: A Survey of Current Theoretical Developments,” Neurocomputing 608 (2024): 128373, 10.1016/j.neucom.2024.128373.

[44] P. Dhariwal and A. Nichol , “Diffusion Models Beat GANs on Image Synthesis,” in Advances in Neural Information Processing Systems 34, eds. M. Ranzato , A. Beygelzimer , Y. Dauphin , P. S. Liang , and J. W. Vaughan , (Neural Information Processing Systems, 2021).

[45] H. Liu , Z. Chen , and Y. Yuan , “AudioLDM: Text‐to‐Audio Generation with Latent Diffusion Models,” in International Conference on Machine Learning , ed. A. Krause , E. Brunskill , K. Cho , B. Engelhardt , S. Sabato , and J. Scarlett , Proceedings of Machine Learning Research (Jmlr‐Journal Machine Learning Research, 2023).

[46] Z. Wang and C. Ventre , “A Financial Time Series Denoiser Based on Diffusion Models,” in Proceedings of the 5th ACM International Conference on AI in Finance (ACM, 2024), 72–80, 10.1145/3677052.3698649.

[47] A. Nichol and P. Dhariwal , “Improved Denoising Diffusion Probabilistic Models,” In Proceedings of the 38th International Conference on Machine Learning ; Proceedings of Machine Learning Research, 139 (2021): 8162–8171.

[48] D. Dematties , C. Wen , M. D. Pérez , D. Zhou , and S.‐L. Zhang , “Deep Learning of Nanopore Sensing Signals Using a Bi‐Path Network,” ACS Nano 15, no. 9 (2021): 14419–14429, 10.1021/acsnano.1c03842. PMC8482760 34583465

[49] S. Choi , Yet‐Another‐Pytorch‐Tutorial‐v2. GitHub, https://github.com/sjchoi86/yet‐another‐pytorch‐tutorial‐v2.

[50] D. Dematties , C. Wen , and S.‐L. Zhang , “A Generalized Transformer‐Based Pulse Detection Algorithm,” ACS Sensors 7, no. 9 (2022): 2710–2720, 10.1021/acssensors.2c01218. PMC9513795 36039873

[51] J. Song , C. Meng , and S. Ermon , “Denoising Diffusion Implicit Models,” In International Conference on Learning Representations (ICLR) , (2021).

[52] L. Dingeldein , P. Cossio , and R. Covino , “Simulation‐Based Inference of Single‐Molecule Force Spectroscopy,” Machine Learning: Science and Technology 4, no. 2 (2023): 025009, 10.1088/2632-2153/acc8b8.

