# Supplementary material for: Denoise Stepwise Signals by Diffusion Model‐Based Approach
Source: Small Methods. 2026 Aug 13;10(18):e70964. doi: 10.1002/smtd.70964 (PMC13599693; doi:10.1002/smtd.70964)
Supplement: Supplementary file 1 [file SMTD-10-e70964-s001.pdf]

Supporting Information

Denoise Stepwise Signals by Diffusion Model Based Approach

Xingdi Tong[1], Chenyu Wen[2*]

1. Department of Civil and Industrial Engineering, Ångströmlaboratoriet, Uppsala University, Lägerhyddsvägen 1, 75237, Uppsala, Sweden

2. Division of Solid-State Electronics, Department of Electrical Engineering, Ångströmlaboratoriet, Uppsala University, Lägerhyddsvägen 1, 75237, Uppsala, Sweden

* Corresponding author: Chenyu Wen: chenyu.wen@angstrom.uu.se

Table of Contents:

Supporting Note 1. Network architecture

Supporting Note 2. Data generation

Supporting Note 3. Generation of noise

Supporting Note 4. Optimization process

Supporting Note 5. Design and implementation of low-pass filters

Supporting Note 6. Design and implementation of Haar wavelet denoising

Supporting Note 7. Design and implementation of HMM

Supporting Note 8. Design and implementation of AutoStepfinder

Supporting Note 9. Design and implementation of TV_Unet

Figure S1. Architecture of the core components in the network

Figure S2. Comparison of processed signals by low-pass filters with different cutoff frequencies in time and frequency domains

Figure S3. Histograms of dwell times of each state of the sm-FRET data

Figure S4. Validation of SSDM on a three-state simulated sm-FRET dataset

Supporting Note 1. Network architecture

We designed a one-dimensional (1D) convolutional neural network (CNN) based on the U-Net architecture to predict noise $\epsilon_\theta(x_t, t)$. This network adopts an encoder-decoder structure and fuses multi-scale features through skip connections. The entire network consists of a four-layer downsampling encoder and a four-layer upsampling decoder, forming a symmetrical structure with a depth of four layers. The input of model is a 1D noisy stepwise signals x_t with shape 1×1000. The embedding vector at diffusion time step t is also fed into the network, enabling the model to perform noise prediction at different noise levels.

In the encoder process, to enhance model capacity, the size of the channels is scaled up from 192 to 384, 768, and 1536. In each layer, the network performs a downsampling on signal, thereby increasing the temporal scope captured by subsequent convolutions. In the final bottleneck layer, the network learns the overall trend of signal. Then, the decoder performs upsampling layer by layer with a symmetric structure as the encoder. Along the flow, it merges the corresponding encoder features into the decoding path through skip connections at each layer. This approach prevents detail loss caused by downsampling, enabling the network to simultaneously learn local fine structures and global general patterns.

The main body of the encoder and decoder consists of multiple ResBlocks. In each ResBlock, local temporal features are first extracted by a 1D convolution with a kernel size of three and padding of one. Then, the SiLU activation function is applied to introduce a nonlinear transformation. Feature maps are subjected to adaptive group normalization, which modulates the normalized features using scale and shift parameters derived from temporal step embeddings. This enables adaptive adjustment of feature representations across different diffusion time steps.

Convolutional structures can efficiently capture local patterns but have limited capacity to model global dependencies across time steps. To address this issue, we adopted a full-scale attention integration strategy, considering the lower computational complexity of 1D signals. In each layer of both the encoder and decoder, an Attention Block was connected after the ResBlock.

To improve the stability of gradient propagation in deep networks, input feature maps $x \in \mathbb{R}^{C\times L}$, where C is the number of channels, and L is the length of feature, first proceed through a group normalization layer. The channel dimension is divided into 16 groups performing independent normalization within each group. The normalized features are expanded to $3C$ channels via a convolutional layer and split into query, Q, key, K, and value, V. To learn information from multiple perspectives, features are divided into four attention heads, each independently processing its sub-features. For each head, compute the dot product between Q and K^{T} to generate correlation matrix QK^T. After Softmax normalization, this matrix serves as weights for aggregating V, enabling each time step to incorporate information from the entire sequence. The aggregated global features proceed through an output projection layer for channel integration. In the output layer, initial weights and biases are set to zero, causing the attention module to behave as an identity mapping during early stage of training. This strategy enables the model to gradually learn and superimpose global residual corrections while preserving local convolutional features.

Supporting Note 2. Data generation

We simulated stepwise signals by a 1D Markov chain. By allocating a fixed amplitude to each state, the segmented constant values of the signal are obtained. State transitions are determined by the transition rate matrix *M*. The diagonal elements m_{ii} of the matrix govern the distribution of state residence times, leading to an average dwell time τ_i determined by m_{ii}, where $\tau_i = 1/|m_{ii}|$. The off-diagonal element m_{ij} represents the rate at which the system transitions from state i to state j. Subsequently, Gaussian pink noise of varying intensities is added to generate the corresponding noisy signals.

To construct noisy stepwise signals, we generate zero-mean Gaussian noise with a controllably designed different RMS level, corresponding to different target SNRs. SNR is defined as the ratio of the spike minimum difference among levels, as amplitude to the peak-to-peak value of the background noise. For Gaussian noise, the peak-to-peak value is typically estimated as six times the root-mean-square value noise. By adjusting different SNR values, we can obtain noise of varying levels. After noise is generated, it will be added to the pre-generated stepwise signals to form noisy signals.

We constructed a balanced dataset comprising three types of single-molecule stepwise signals: 2-state, 3-state, and 4-state. All signals were generated at a sampling rate of 1 Hz, with each fixed at 1000 time points. A total of 10,800 signals samples were generated for training and optimizing. To keep the data simple, state value of all signals is defined using a unified normalization method. The state levels uniformly distributed in the normalized interval [0, 1]. In other words, the 2-state signal uses state values of possesses two levels located at 0 and 1. The 3-state signal has three levels at 0, 0.5, and 1, and the 4-state signal has four levels at 0, 0.33, 0.66, and 1.

To achieve a gradual progression in dynamic complexity and recognition difficulty across different signal types while ensuring comprehensive coverage of the parameter space, we designed tailored generation logic for signal type. For the 2-state signals, eight representative combinations of transition rates were selected, as shown in Table S1. For the 3-state signals, the transition rate matrix contains more independent parameters. First, we set the escape rates of the three states, m_{ii}, i = 1, 2, and 3, to be identical, which is divided into six rate categories, ranging from extremely slow (0.01), slow (0.05), moderately slow (0.1), moderate (0.3), fairly fast (0.5), to fast (0.8). Subsequently, within each rate group, two representative internal transition modes were selected. By combining six escape rate types with two internal modes, 12 typical 3-state transition rate matrices were ultimately obtained, as shown in Table S1. In the 4-state signals, the escape rate of one reference state was fixed as an anchor point, while the escape rates of the other three states were varied around it. 16 representatives transition rate matrices were generated, as shown in Table S1. Each matrix generated 100 samples, and covering SNR = 1, SNR = 3, and SNR = 5 types. Therefore, there are 2400, 3600, and 4,800 samples for 2-, 3-, and 4-state signals, respectively.

Table S1. Signal Parameter for train dataset

State Number	Transition rate matrix (M)	State Number	Transition rate matrix (M)	State Number	Transition rate matrix (M)
	$\begin{bmatrix} -0.01 & 0.01 \\ 0.01 & -0.01 \end{bmatrix}$		$\begin{bmatrix} -0.01 & 0.005 & 0.005 \\ 0.005 & -0.01 & 0.005 \\ 0.005 & 0.005 & -0.01 \end{bmatrix}$		$\begin{bmatrix} -0.05 & 0.01 & 0.02 & 0.02 \\ 0.015 & -0.05 & 0.015 & 0.02 \\ 0.012 & 0.018 & -0.05 & 0.02 \\ 0.019 & 0.011 & 0.02 & -0.05 \end{bmatrix}$
2		3	$\begin{bmatrix} -0.01 & 0.002 & 0.008 \\ 0.002 & -0.01 & 0.008 \\ 0.002 & 0.008 & -0.01 \end{bmatrix}$	4	$\begin{bmatrix} -0.05 & 0.01 & 0.02 & 0.02 \\ 0.15 & -0.35 & 0.05 & 0.15 \\ 0.012 & 0.018 & -0.05 & 0.02 \\ 0.019 & 0.011 & 0.02 & -0.05 \end{bmatrix}$
	$\begin{bmatrix} -0.01 & 0.01 \\ 0.02 & -0.02 \end{bmatrix}$		$\begin{bmatrix} -0.05 & 0.02 & 0.03 \\ 0.02 & -0.05 & 0.03 \\ 0.02 & 0.03 & -0.05 \end{bmatrix}$		$\begin{bmatrix} -0.05 & 0.01 & 0.02 & 0.02 \\ 0.005 & -0.05 & 0.025 & 0.02 \\ 0.12 & 0.18 & -0.5 & 0.2 \\ 0.019 & 0.011 & 0.02 & -0.05 \end{bmatrix}$

$\begin{bmatrix} -0.01 & 0.01 \\ 0.05 & -0.05 \end{bmatrix}$	$\begin{bmatrix} -0.05 & 0.04 & 0.01 \\ 0.04 & -0.05 & 0.01 \\ 0.04 & 0.01 & -0.05 \end{bmatrix}$<br>$\begin{bmatrix} -0.1 & 0.05 & 0.05 \\ 0.05 & -0.1 & 0.05 \\ 0.05 & 0.05 & -0.1 \end{bmatrix}$	$\begin{bmatrix} -0.05 & 0.01 & 0.02 & 0.02 \\ 0.005 & -0.05 & 0.025 & 0.02 \\ 0.012 & 0.018 & -0.05 & 0.02 \\ 0.06 & 0.01 & 0.03 & -0.1 \end{bmatrix}$<br>$\begin{bmatrix} -0.1 & 0.03 & 0.04 & 0.03 \\ 0.05 & -0.1 & 0.02 & 0.03 \\ 0.03 & 0.03 & -0.1 & 0.04 \\ 0.05 & 0.02 & 0.03 & -0.1 \end{bmatrix}$<br>$\begin{bmatrix} -0.1 & 0.03 & 0.04 & 0.03 \\ 0.1 & -0.3 & 0.1 & 0.1 \\ 0.1 & 0.2 & -0.5 & 0.2 \\ 0.019 & 0.011 & 0.02 & -0.05 \end{bmatrix}$
$\begin{bmatrix} -0.01 & 0.01 \\ 0.1 & -0.1 \end{bmatrix}$	$\begin{bmatrix} -0.1 & 0.01 & 0.09 \\ 0.01 & -0.1 & 0.09 \\ 0.01 & 0.09 & -0.1 \end{bmatrix}$	$\begin{bmatrix} -0.1 & 0.03 & 0.04 & 0.03 \\ 20 & -50 & 20 & 10 \\ 0.1 & 0.2 & -0.5 & 0.2 \\ 0.019 & 0.011 & 0.02 & -0.05 \end{bmatrix}$<br>$\begin{bmatrix} -0.1 & 0.03 & 0.04 & 0.03 \\ 20 & -50 & 20 & 10 \\ 30 & 20 & -60 & 10 \\ 0.019 & 0.011 & 0.02 & -0.05 \end{bmatrix}$
$\begin{bmatrix} -0.01 & 0.01 \\ 0.5 & -0.5 \end{bmatrix}$	$\begin{bmatrix} -0.3 & 0.1 & 0.2 \\ 0.1 & -0.3 & 0.2 \\ 0.1 & 0.2 & -0.3 \end{bmatrix}$<br>$\begin{bmatrix} -0.3 & 0.05 & 0.25 \\ 0.05 & -0.3 & 0.25 \\ 0.05 & 0.25 & -0.3 \end{bmatrix}$	$\begin{bmatrix} -0.3 & 0.07 & 0.11 & 0.12 \\ 0.1 & -0.3 & 0.1 & 0.1 \\ 0.08 & 0.12 & -0.3 & 0.1 \\ 0.09 & 0.08 & 0.13 & -0.3 \end{bmatrix}$<br>$\begin{bmatrix} -0.3 & 0.07 & 0.11 & 0.12 \\ 0.07 & -0.3 & 0.11 & 0.12 \\ 0.05 & 0.05 & -0.3 & 0.2 \\ 0.8 & 0.2 & 0.5 & -0.15 \end{bmatrix}$
$\begin{bmatrix} -0.05 & 0.05 \\ 0.01 & -0.01 \end{bmatrix}$	$\begin{bmatrix} -0.5 & 0.2 & 0.3 \\ 0.2 & -0.5 & 0.3 \\ 0.2 & 0.3 & -0.5 \end{bmatrix}$	$\begin{bmatrix} -0.3 & 0.07 & 0.11 & 0.12 \\ 0.2 & -0.8 & 0.3 & 0.3 \\ 0.07 & 0.11 & -0.3 & 0.12 \\ 0.07 & 0.12 & 0.11 & -0.3 \end{bmatrix}$<br>$\begin{bmatrix} -0.3 & 0.07 & 0.11 & 0.12 \\ 0.07 & -0.3 & 0.11 & 0.12 \\ 0.5 & 0.05 & -0.1 & 0.25 \\ 0.07 & 0.12 & 0.11 & -0.3 \end{bmatrix}$
$\begin{bmatrix} -0.2 & 0.2 \\ 0.01 & -0.01 \end{bmatrix}$	$\begin{bmatrix} -0.5 & 0.1 & 0.4 \\ 0.1 & -0.5 & 0.4 \\ 0.1 & 0.4 & -0.5 \end{bmatrix}$<br>$\begin{bmatrix} -0.8 & 0.4 & 0.4 \\ 0.4 & -0.8 & 0.4 \\ 0.4 & 0.4 & -0.8 \end{bmatrix}$	$\begin{bmatrix} -0.5 & 0.1 & 0.2 & 0.2 \\ 0.15 & -0.5 & 0.15 & 0.2 \\ 0.12 & 0.18 & -0.5 & 0.2 \\ 0.19 & 0.11 & 0.2 & -0.5 \end{bmatrix}$<br>$\begin{bmatrix} -0.5 & 0.1 & 0.2 & 0.2 \\ 0.015 & -0.1 & 0.015 & 0.07 \\ 0.12 & 0.18 & -0.5 & 0.2 \\ 0.2 & 0.3 & 0.5 & -1 \end{bmatrix}$
$\begin{bmatrix} -0.5 & 0.5 \\ 0.01 & -0.01 \end{bmatrix}$	$\begin{bmatrix} -0.8 & 0.1 & 0.7 \\ 0.1 & -0.8 & 0.7 \\ 0.1 & 0.7 & -0.8 \end{bmatrix}$	$\begin{bmatrix} -0.5 & 0.1 & 0.2 & 0.2 \\ 0.015 & -0.1 & 0.015 & 0.07 \\ 0.12 & 0.18 & -0.5 & 0.2 \\ 0.19 & 0.11 & 0.2 & -0.5 \end{bmatrix}$<br>$\begin{bmatrix} -10 & 3 & 4 & 3 \\ 0.2 & -0.5 & 0.2 & 0.1 \\ 0.3 & 0.2 & -0.6 & 0.1 \\ 19 & 11 & 20 & -50 \end{bmatrix}$

The test dataset was generated independently from the training dataset to evaluate the generalization ability and robustness of SSDM. Compared with the training data, the test dataset mainly consists of signals with

more rapid state transitions, corresponding to higher escape rates, as shown in the Table S2. Within the whole test dataset, a total of 40 distinct parameter combinations were included, comprising 8 for 2-state signals, 16 for 3-state signals, and 16 for 4-state signals. Each combination contains 20 samples for each SNR level (SNR = 0.25, 0.5, 1, 3, and 5), resulting in 4,000 samples per SNR level and 20,000 samples in total.

Table S2. Signal Parameter for test dataset

State Number	Transition rate matrix (M)	State Number	Transition rate matrix (M)	State Number	Transition rate matrix (M)
2	$\begin{bmatrix} -0.1 & 0.1 \\ 0.5 & -0.5 \end{bmatrix}$	3	$\begin{bmatrix} -0.1 & 0.05 & 0.05 \\ 0.05 & -0.1 & 0.05 \\ 0.05 & 0.05 & -0.1 \end{bmatrix}$	4	$\begin{bmatrix} -0.1 & 0.03 & 0.04 & 0.03 \\ 0.03 & -0.1 & 0.04 & 0.03 \\ 0.03 & 0.04 & -0.1 & 0.03 \\ 0.03 & 0.04 & 0.03 & -0.1 \end{bmatrix}$
			$\begin{bmatrix} -0.1 & 0.05 & 0.05 \\ 0.05 & -0.1 & 0.05 \\ 0.1 & 0.2 & -0.3 \end{bmatrix}$		$\begin{bmatrix} -0.1 & 0.03 & 0.04 & 0.03 \\ 0.03 & -0.1 & 0.04 & 0.03 \\ 0.03 & 0.04 & -0.1 & 0.03 \\ 0.02 & 0.01 & 0.01 & -0.04 \end{bmatrix}$
	$\begin{bmatrix} -0.1 & 0.1 \\ 0.8 & -0.8 \end{bmatrix}$		$\begin{bmatrix} -0.1 & 0.05 & 0.05 \\ 0.05 & -0.1 & 0.05 \\ 0.2 & 0.3 & -0.5 \end{bmatrix}$		$\begin{bmatrix} -0.1 & 0.03 & 0.04 & 0.03 \\ 0.03 & -0.1 & 0.04 & 0.03 \\ 0.03 & 0.04 & -0.1 & 0.03 \\ 0.02 & 0.02 & 0.01 & -0.05 \end{bmatrix}$
			$\begin{bmatrix} -0.1 & 0.05 & 0.05 \\ 0.05 & -0.1 & 0.05 \\ 0.04 & 0.01 & -0.05 \end{bmatrix}$		$\begin{bmatrix} -0.1 & 0.03 & 0.04 & 0.03 \\ 0.03 & -0.1 & 0.04 & 0.03 \\ 0.03 & 0.04 & -0.1 & 0.03 \\ 0.06 & 0.07 & 0.07 & -0.2 \end{bmatrix}$
	$\begin{bmatrix} -0.2 & 0.2 \\ 0.5 & -0.5 \end{bmatrix}$		$\begin{bmatrix} -0.3 & 0.1 & 0.2 \\ 0.1 & -0.3 & 0.2 \\ 0.05 & 0.05 & -0.1 \end{bmatrix}$		$\begin{bmatrix} -0.125 & 0.04 & 0.04 & 0.045 \\ 0.04 & -0.125 & 0.04 & 0.045 \\ 0.04 & 0.04 & -0.125 & 0.045 \\ 0.03 & 0.04 & 0.03 & -0.1 \end{bmatrix}$
			$\begin{bmatrix} -0.3 & 0.1 & 0.2 \\ 0.1 & -0.3 & 0.2 \\ 0.1 & 0.2 & -0.3 \end{bmatrix}$		$\begin{bmatrix} -0.125 & 0.04 & 0.04 & 0.045 \\ 0.04 & -0.125 & 0.04 & 0.045 \\ 0.04 & 0.04 & -0.125 & 0.045 \\ 0.02 & 0.02 & 0.01 & -0.05 \end{bmatrix}$
	$\begin{bmatrix} -0.2 & 0.2 \\ 0.8 & -0.8 \end{bmatrix}$		$\begin{bmatrix} -0.3 & 0.1 & 0.2 \\ 0.1 & -0.3 & 0.2 \\ 0.2 & 0.3 & -0.5 \end{bmatrix}$		$\begin{bmatrix} -0.125 & 0.04 & 0.04 & 0.045 \\ 0.04 & -0.125 & 0.04 & 0.045 \\ 0.04 & 0.04 & -0.125 & 0.045 \\ 0.02 & 0.01 & 0.01 & -0.04 \end{bmatrix}$
			$\begin{bmatrix} -0.3 & 0.1 & 0.2 \\ 0.1 & -0.3 & 0.2 \\ 0.04 & 0.01 & -0.05 \end{bmatrix}$		$\begin{bmatrix} -0.125 & 0.04 & 0.04 & 0.045 \\ 0.04 & -0.125 & 0.04 & 0.045 \\ 0.04 & 0.04 & -0.125 & 0.045 \\ 0.06 & 0.07 & 0.07 & -0.2 \end{bmatrix}$
	$\begin{bmatrix} -0.5 & 0.5 \\ 0.5 & -0.5 \end{bmatrix}$		$\begin{bmatrix} -0.5 & 0.2 & 0.3 \\ 0.2 & -0.5 & 0.3 \\ 0.05 & 0.05 & -0.1 \end{bmatrix}$		$\begin{bmatrix} -0.2 & 0.06 & 0.07 & 0.07 \\ 0.06 & -0.2 & 0.07 & 0.07 \\ 0.06 & 0.07 & -0.2 & 0.07 \\ 0.03 & 0.04 & 0.03 & -0.1 \end{bmatrix}$
			$\begin{bmatrix} -0.5 & 0.2 & 0.3 \\ 0.2 & -0.5 & 0.3 \\ 0.1 & 0.2 & -0.3 \end{bmatrix}$		$\begin{bmatrix} -0.2 & 0.06 & 0.07 & 0.07 \\ 0.06 & -0.2 & 0.07 & 0.07 \\ 0.06 & 0.07 & -0.2 & 0.07 \\ 0.02 & 0.02 & 0.01 & -0.05 \end{bmatrix}$
	$\begin{bmatrix} -0.5 & 0.5 \\ 0.8 & -0.8 \end{bmatrix}$		$\begin{bmatrix} -0.5 & 0.2 & 0.3 \\ 0.2 & -0.5 & 0.3 \\ 0.2 & 0.3 & -0.5 \end{bmatrix}$		$\begin{bmatrix} -0.2 & 0.06 & 0.07 & 0.07 \\ 0.06 & -0.2 & 0.07 & 0.07 \\ 0.06 & 0.07 & -0.2 & 0.07 \\ 0.02 & 0.01 & 0.01 & -0.04 \end{bmatrix}$

	$\begin{bmatrix}-0.5 & 0.2 & 0.3\\ 0.2 & -0.5 & 0.3\\ 0.04 & 0.01 & -0.05\end{bmatrix}$	$\begin{bmatrix}-0.2 & 0.06 & 0.07 & 0.07\\ 0.06 & -0.2 & 0.07 & 0.07\\ 0.06 & 0.07 & -0.2 & 0.07\\ 0.06 & 0.07 & 0.07 & -0.2\end{bmatrix}$
$\begin{bmatrix}-0.8 & 0.8\\ 0.5 & -0.5\end{bmatrix}$	$\begin{bmatrix}-0.05 & 0.04 & 0.01\\ 0.04 & -0.05 & 0.01\\ 0.05 & 0.05 & -0.1\end{bmatrix}$	$\begin{bmatrix}-0.5 & 0.2 & 0.2 & 0.1\\ 0.2 & -0.5 & 0.2 & 0.1\\ 0.2 & 0.2 & -0.5 & 0.1\\ 0.03 & 0.04 & 0.03 & -0.1\end{bmatrix}$
	$\begin{bmatrix}-0.05 & 0.04 & 0.01\\ 0.04 & -0.05 & 0.01\\ 0.1 & 0.2 & -0.3\end{bmatrix}$	$\begin{bmatrix}-0.5 & 0.2 & 0.2 & 0.1\\ 0.2 & -0.5 & 0.2 & 0.1\\ 0.2 & 0.2 & -0.5 & 0.1\\ 0.02 & 0.02 & 0.01 & -0.05\end{bmatrix}$
$\begin{bmatrix}-0.8 & 0.8\\ 0.8 & -0.8\end{bmatrix}$	$\begin{bmatrix}-0.05 & 0.04 & 0.01\\ 0.04 & -0.05 & 0.01\\ 0.2 & 0.3 & -0.5\end{bmatrix}$	$\begin{bmatrix}-0.5 & 0.2 & 0.2 & 0.1\\ 0.2 & -0.5 & 0.2 & 0.1\\ 0.2 & 0.2 & -0.5 & 0.1\\ 0.02 & 0.01 & 0.01 & -0.04\end{bmatrix}$
	$\begin{bmatrix}-0.05 & 0.04 & 0.01\\ 0.04 & -0.05 & 0.01\\ 0.04 & 0.01 & -0.05\end{bmatrix}$	$\begin{bmatrix}-0.5 & 0.2 & 0.2 & 0.1\\ 0.2 & -0.5 & 0.2 & 0.1\\ 0.2 & 0.2 & -0.5 & 0.1\\ 0.06 & 0.07 & 0.07 & -0.2\end{bmatrix}$

Supporting Note 3. Generation of noise

To evaluate the robustness of the denoising model under different noise characteristics, both Gaussian white noise and pink noise with varying intensities were added to the simulated signals. Gaussian white noise with a flat power spectral density (PSD) $S(f) = \sigma^2$, where σ^2 is the noise variance. For pink noise, the PSD follows $S(f) \propto 1/f$, where f is the frequency. Gaussian white noise was used as a baseline noise model to represent random measurement noise commonly encountered in signal acquisition. In contrast, pink noise includes low-frequency components that are often present in experimental single-molecule measurements due to instrumental drift and environmental effects. By incorporating both noise types, the simulations cover a broader range of noise types, enabling a more comprehensive assessment of model performance under different conditions. Importantly, the training of SSDM uses only Gaussian white noise which is added through the forward add noise process, while the test datasets additionally include signals corrupted by pink noise. This setup constitutes a natural assessment of the robustness of SSDM under train-test noise distribution mismatch, where the model is evaluated on noise types not encountered during training.

Supporting Note 4. Optimization process

To enhance the generalization capabilities of deep neural networks, we employed the *AdamW* optimizer during training with a weight decay of 1×10^{-4}. We also introduced the Cosine Annealing Scheduler to address the dual requirements of rapid convergence and refined search. The learning rate, as a hyperparameter, will be incorporated into subsequent search optimization. We introduced the cosine annealing scheduler to balance rapid convergence with finer search capabilities. The learning rate decays along a cosine curve based on epoch count, enabling the model to refine weights near local minima during late training phases while avoiding oscillations around optimal solutions. This learning rate will be incorporated as a hyperparameter into search optimization.

This study employed an automated Bayesian optimization strategy using the *Weights & Biases (W&B)* sweep framework. Our search objective is to maximize the *Score* value. This metric aims to find a balance point in model performance, maximizing state transition detection accuracy as indicated by a higher *F1*, while simultaneously minimizing signal reconstruction error as reflected by a lower *MSE*. Based on this objective function, the optimization algorithm performed 15 rounds of iterative searches within the predefined hyperparameter space, ultimately identifying the optimal parameter combination.

In each iteration, 5-fold cross-validation is performed on the current set of hyperparameters. This involves dividing the training data into five folds, training the model using four folds in each fold, calculating the score metric on the remaining fold, and finally aggregating the average Score across all folds as the score for that set of hyperparameters. The newly obtained hyperparameter combinations and their corresponding Score are fed back to the Bayesian optimization algorithm to update the model's understanding of the objective function.

The model was ultimately trained for 150 epochs with a batch size of 16. The initial learning rate is set to 7.61×10^{-5} and is smoothly decayed to 1×10^{-6} using cosine annealing strategy. The network is constructed based on 192 channels, employing channel scaling factors (1, 2, 4, 8) to build a 4-layer U-Net architecture, and incorporates a 4-head full-scale self-attention mechanism. In the final loss function, the amplitude weight $\lambda_{amp} = 14.53$ and the edge weight $\lambda_{edge} = 8.95$.

Supporting Note 5. Design and implementation of low-pass filters

We employed a 4-order low-pass filter to perform denoising on the signal. To more effectively compare the denoising performance of SSDM, we designed a parameter adaptation mechanism based on grid search to determine the optimal cutoff frequency, f_s. We have predefined certain key candidate cutoff frequencies based on the physical features of the signal (e.g., f_s = [0.01, 0.08]). These values represent different levels of filtering intensity. Lower frequencies represent stronger smoothing effects, but may also smooth out signal details. Higher frequencies preserve more detail, but also retain more noise. Then the iterative phase begins, denoising with each candidate cutoff frequency in sequence. By evaluating the performance of each candidate cutoff frequency, we select the filter most suitable for the current signal. Regarding the evaluation, we employ the same method as SSDM. Using the same thresholding method to determine the transition points after denoising,

we then calculate *F1*, *MSE*, and the *Score*. By comparing these results, we select the cutoff frequency with the highest *Score* as the cutoff frequency for the filter applied to the current signal.

Supporting Note 6. Design and implementation of Haar wavelet denoising

The Haar wavelet–based denoising was implemented using the discrete wavelet transform with the Haar basis. Each input noisy signal was decomposed into approximation and detail coefficients across 3 decomposition levels. Soft thresholding was applied to the detail coefficients at each level, with the threshold determined by the universal threshold rule:

$$\lambda = \sigma\sqrt{2\ln N} \tag{S1}$$

Where σ is the standard deviation of the noise estimated from the finest-level detail coefficients via the median absolute deviation, and N is the signal length. The denoised signal was then reconstructed by the inverse discrete wavelet transform. Transition points and quantitative metrics (e.g. *MSE*, *F1* and *Score*) were computed using the same evaluation procedure as for SSDM.

Supporting Note 7. Design and implementation of HMM

We build an HMM model based on the BIC criterion to determine the number of signal states. However, since HMM is not a tool specifically designed for denoising, we adapt the HMM to better compare its denoising performance with that of SSDM. We define a candidate state set $S = \{2, 3, 4, 5, 6\}$, assuming the number of signal states belongs to the entire state set. The HMM model uses the number of states in each state to fit the current signal. During each fitting process, the HMM learns parameters corresponding to each state count, including the mean and state transition probabilities. We then utilize the BIC criterion to automatically select the optimal number of states. According to BIC = $-2\ln(L) + k\ln(n)$, where L represents the maximum value of the likelihood function. The calculation of $\ln(L)$ employs the forward algorithm. This algorithm accumulates the sum of probabilities for all possible hidden state paths that generate the current observed data through dynamic programming. k represents the total number of free parameters to be estimated in the model, and n is the total number of data points observed. Ultimately, the model with the smallest BIC value will be selected as the optimal model. Then, the optimal model is used to predict the state for the entire signal segment. The model assigns the most probable hidden state to each time point and replaces the signal value at that point with the mean of the corresponding state, thereby obtaining the denoised signal. We employ the same evaluation method as SSDM. By identifying transition points in the signal using the thresholding method, we calculate *F1*, *MSE*, and the *Score*. This enables direct comparison of the denoising results with SSDM through the *Score* value.

Supporting Note 8. Design and implementation of Autostepfinder

AutoStepfinder was implemented following the algorithm described by Loeff *et al.*[1]. The algorithm iteratively detects stepwise transitions in the noisy signal by minimizing the residual between data and a piecewise-constant fit, with the iteration terminated when no further significant steps are detected. The key parameter controlling this termination is the acceptance ratio threshold (SMaxThreshold in the GUI), which governs the trade-off between noise suppression and over-segmentation. In our benchmark, the default acceptance ratio threshold could not generalize across the wide SNR range (0.25 to 5) of the test dataset. We therefore manually adjusted the acceptance ratio threshold for different signal quality conditions, based on visual inspection of the resulting denoised traces. Specifically, for signals with relatively high SNR, the default acceptance ratio was retained or used with minor adjustments, whereas for signals with low SNR, the threshold was lowered to allow the algorithm to detect transitions that would otherwise be rejected as insignificant. This manual tuning was performed for each noise condition to ensure that AutoStepfinder achieved its best achievable performance on each test signal, providing a fair comparison with SSDM.

After threshold selection, the denoised output was exported and imported into our evaluation pipeline. Transition points and quantitative metrics (*F1*, *MSE*, *Score*) were then computed using the same evaluation framework as for SSDM. We note that the requirement of manual parameter tuning per signal condition limits

the practicality of AutoStepfinder for large-scale automated processing, which represents an additional limitation of this method compared to learning-based approaches such as SSDM.

Supporting Note 9. Design and implementation of TV_Unet

TV_Unet is a 1D convolutional autoencoder used as a non-diffusion baseline for comparing with SSDM. The network adopts exactly the same U-Net architecture as SSDM, refer to Supporting Note 1, including the encoder-decoder structure, residual blocks, attention blocks, skip connections, and the multi-scale channel scaling. TV_Unet was trained on the exact same dataset comprising 10,800 paired noisy and noise-free signals as SSDM. To optimize the network for stepwise dynamics, the training objective was formulated as a combination of a Smooth L1 loss and a Total Variation (TV) regularization term to explicitly encourage piecewise-constant outputs:

$$L_{\mathrm{TV}} = L_{\mathrm{Smooth\ L1}}(\widehat{x_0}, x_0) + \lambda_{\mathrm{TV}} \cdot \frac{1}{N-1} \sum_{i=1}^{N-1} \left| \widehat{x_{0,i+1}} - \widehat{x_{0,i}} \right| \quad \text{(S2)}$$

where $\widehat{x_0}$ is the predicted noise-free signal, x_0 is the ground truth, N is the signal length, and the penalty factor λ_{TV} was set to 1.0. The network was trained using the same optimizer (Adam*W*), learning rate schedule (cosine annealing), and total training epochs (150) as SSDM. During inference, the noisy input was directly mapped to a noise-free signal estimate through a single forward pass. This identical architecture and identical dataset baseline definitively isolate and highlight the fundamental performance contribution of the diffusion mechanismin SSDM.

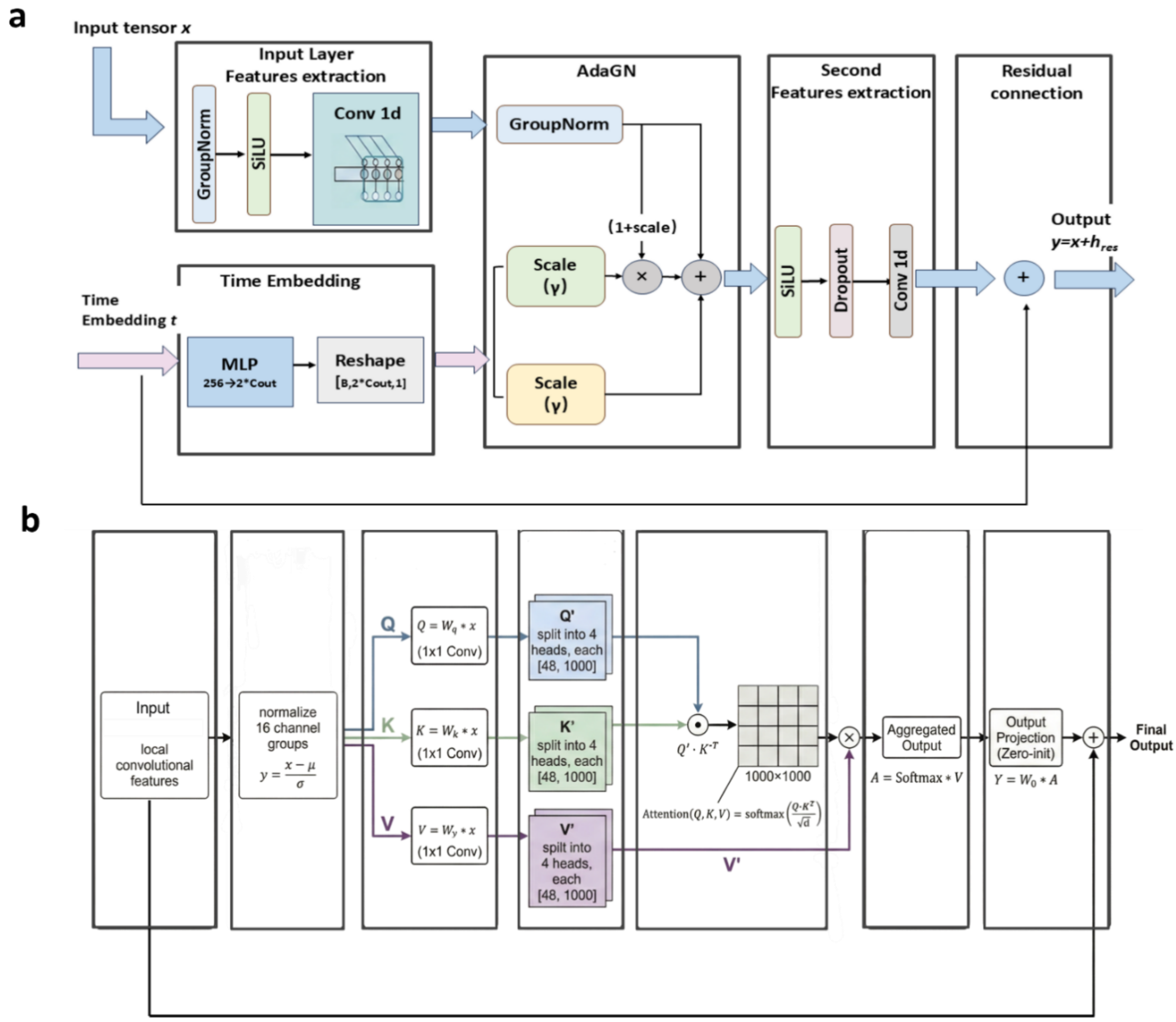

Figure S1. Architecture of the core components in the network. (a) Architecture of residual blocks in the network. (b) Architecture of attention blocks in the network.

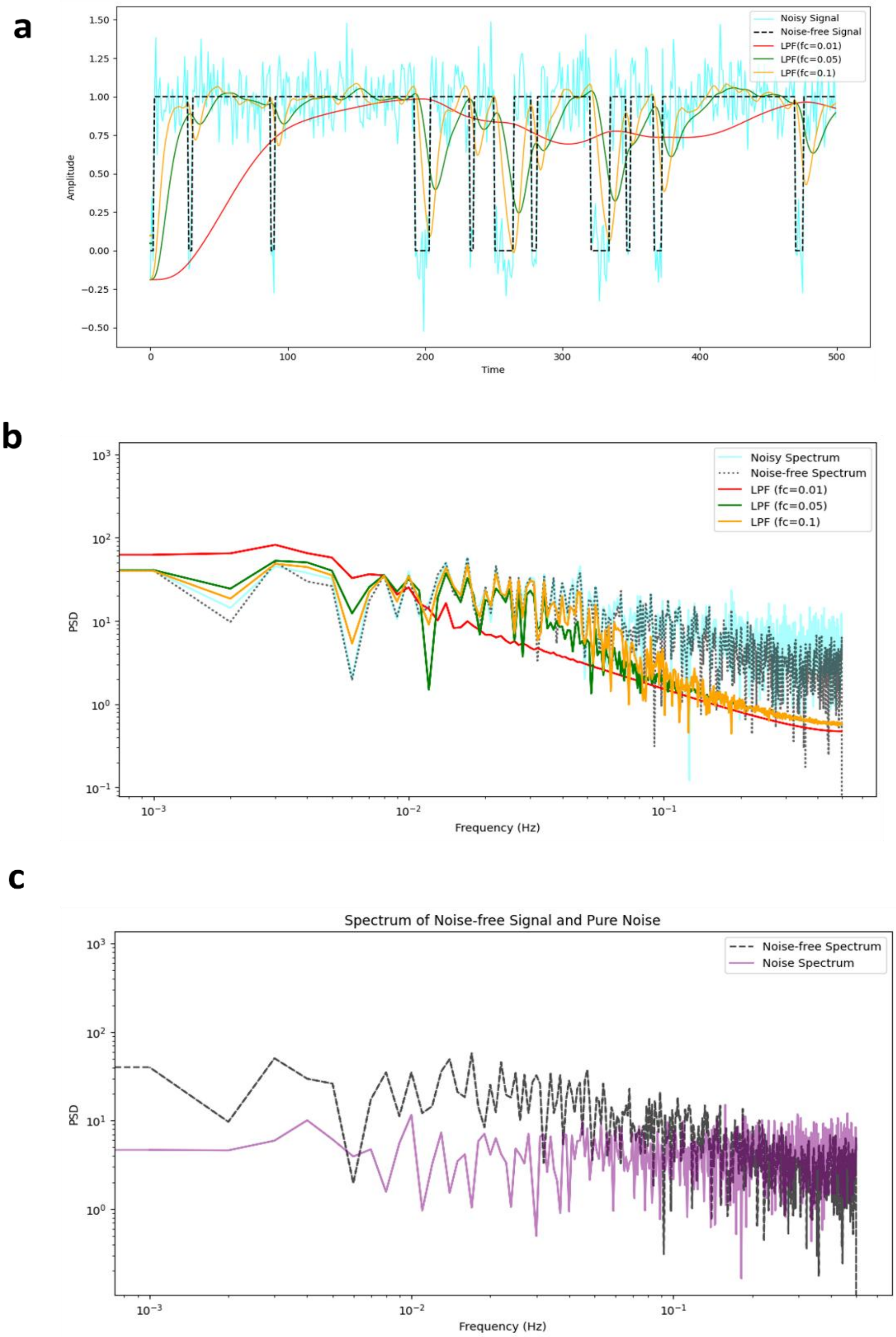

Figure S2. Comparison of processed signals by low-pass filters with different cutoff frequencies in time and frequency domains. (a) Time-domain plot of the noisy signal, the original noise-free signal, and the filtered outputs with cutoff frequencies f_c = 0.01 Hz, 0.05 Hz, and 0.1 Hz. The sampling frequency of the signal is (normalized to) 1 Hz. (b) Spectra of the aforementioned signal traces. It can be clearly seen that the noise-free signal contains abundant high frequency components which is filtered out by low-pass filters. (c) Spectra of noise-free signal and pure noise. We can see that their spectra are highly overlapped, especially when $f > 0.1$ Hz, which makes the directionless filtering impossible.

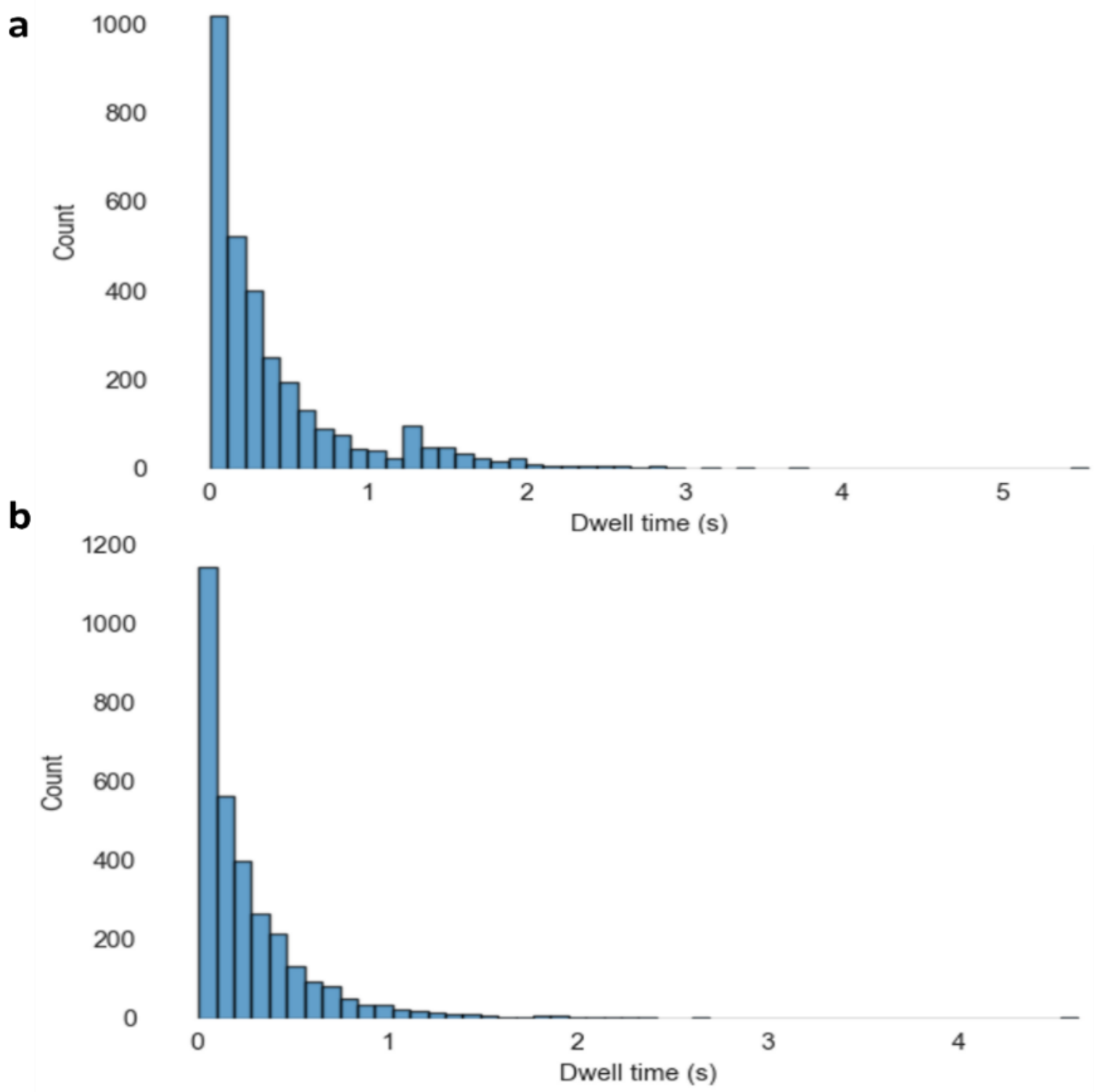

Figure S3. Histogram of dwell times of each state of the sm-FRET data. (a) Histogram of dwell times of state 1 in the sm-FRET signal. (b) Histogram of dwell times of state 2 in the sm-FRET signal.

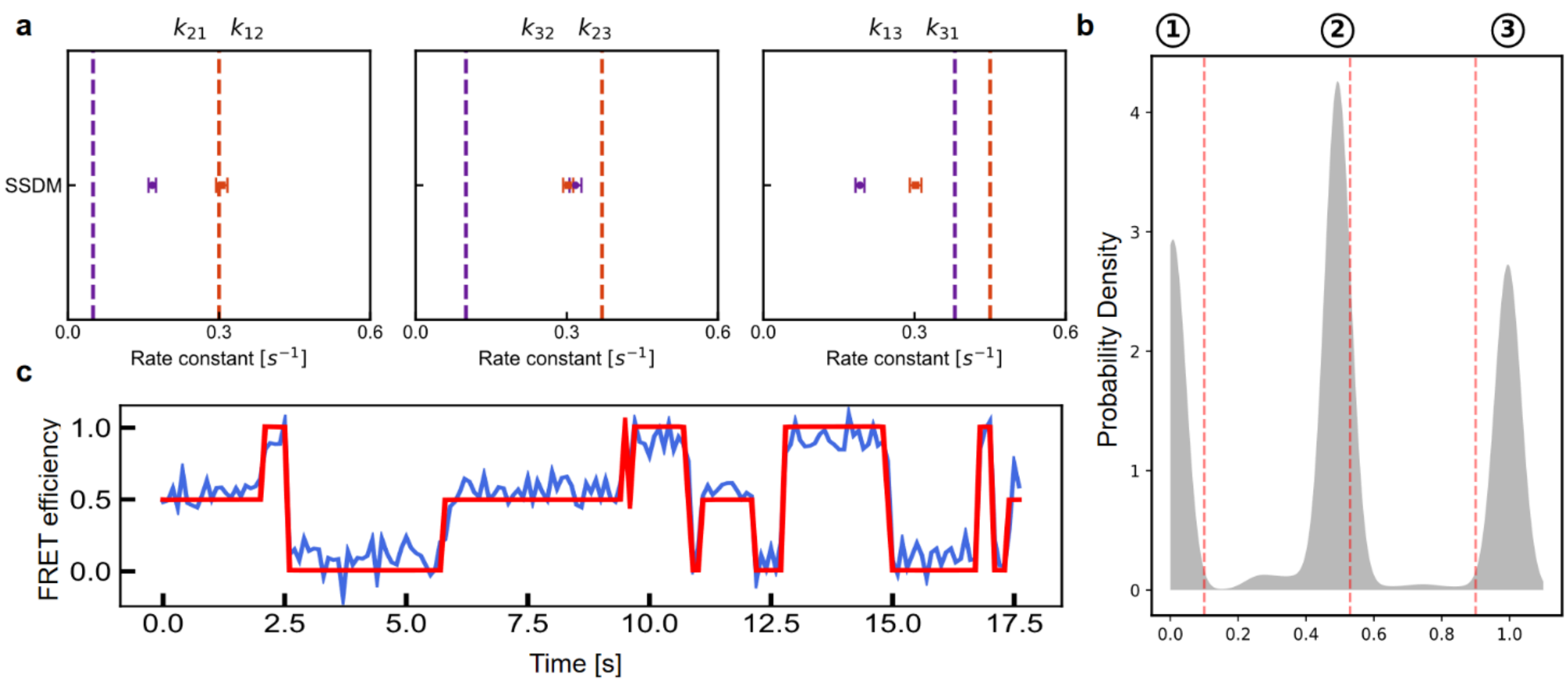

Figure S4. Validation of SSDM on a three-state simulated sm-FRET dataset. (a) Comparison of the six inter-state kinetic rate constants (k_{12}, k_{21}, k_{23}, k_{32}, k_{13}, k_{31}) extracted from SSDM denoised signals with the corresponding ground-truth values. Dashed lines indicate the ground-truth rate constants, and the points with error bars represent the values extracted from SSDM denoised signals. (b) Probability density distribution of FRET efficiency from SSDM denoised signals. The three well-separated peaks correspond to the three FRET states. The red dashed lines represent the ground-truth state levels at 0.1, 0.53, and 0.9. (c) Representative denoised trajectory. The blue curve is the original noisy FRET trajectory, and the red curve is the SSDM-denoised result. The analysis is based on 150 simulated signals (n = 150).

References

(1) Loeff, L.; Kerssemakers, J. W. J.; Joo, C.; Dekker, C. AutoStepfinder: A Fast and Automated Step Detection Method for Single-Molecule Analysis. *Patterns* **2021**, *2* (5), 100256. https://doi.org/10.1016/j.patter.2021.100256.
